# Hyperactive KRAS/MAPK signaling disrupts normal lymphatic vessel architecture and function

**DOI:** 10.3389/fcell.2023.1276333

**Published:** 2023-09-25

**Authors:** Lorenzo M. Fernandes, Jeffrey Tresemer, Jing Zhang, Jonathan J. Rios, Joshua P. Scallan, Michael T. Dellinger

**Affiliations:** ^1^ Hamon Center for Therapeutic Oncology Research, UT Southwestern Medical Center, Dallas, TX, United States; ^2^ McArdle Laboratory for Cancer Research, University of Wisconsin-Madison, Madison, WI, United States; ^3^ Center for Pediatric Bone Biology and Translational Research, Scottish Rite for Children, Dallas, TX, United States; ^4^ McDermott Center for Human Growth and Development, Dallas, TX, United States; ^5^ Department of Molecular Pharmacology and Physiology, Morsani College of Medicine, University of South Florida, Tampa, FL, United States; ^6^ Department of Surgery, UT Southwestern Medical Center, Dallas, TX, United States

**Keywords:** complex lymphatic anomaly, KRAS, trametinib, lymphangiogenesis, lymphatic malformation, Gorham-Stout disease

## Abstract

Complex lymphatic anomalies (CLAs) are sporadically occurring diseases caused by the maldevelopment of lymphatic vessels. We and others recently reported that somatic activating mutations in *KRAS* can cause CLAs. However, the mechanisms by which activating *KRAS* mutations cause CLAs are poorly understood. Here, we show that KRAS^G12D^ expression in lymphatic endothelial cells (LECs) during embryonic development impairs the formation of lymphovenous valves and causes the enlargement of lymphatic vessels. We demonstrate that KRAS^G12D^ expression in primary human LECs induces cell spindling, proliferation, and migration. It also increases AKT and ERK1/2 phosphorylation and decreases the expression of genes that regulate the maturation of lymphatic vessels. We show that MEK1/2 inhibition with the FDA-approved drug trametinib suppresses KRAS^G12D^-induced morphological changes, proliferation, and migration. Trametinib also decreases ERK1/2 phosphorylation and increases the expression of genes that regulate the maturation of lymphatic vessels. We also show that trametinib and Cre-mediated expression of a dominant-negative form of MEK1 (*Map2k1* ^*K97M*^) suppresses KRAS^G12D^-induced lymphatic vessel hyperplasia in embryos. Last, we demonstrate that conditional knockout of wild-type *Kras* in LECs does not affect the formation or function of lymphatic vessels. Together, our data indicate that KRAS/MAPK signaling must be tightly regulated during embryonic development for the proper development of lymphatic vessels and further support the testing of MEK1/2 inhibitors for treating CLAs.

## Introduction

Complex lymphatic anomalies (CLAs) are sporadically occurring diseases caused by the maldevelopment of lymphatic vessels (Iacobas et al., 2020). CLAs include Gorham-Stout disease, generalized lymphatic anomaly, kaposiform lymphangiomatosis, and central conducting lymphatic anomaly (Iacobas et al., 2020). CLA patients can have tortuous dilated lymphatic vessels, multifocal lymphatic malformations (LMs) that infiltrate surrounding tissue, and ectopic lymphatic vessels in bone (Lala et al., 2013; Dellinger et al., 2014; Trenor and Chaudry, 2014; Andreoti et al., 2023). Patients exhibit a wide variety of painful and fatal complications depending on the location of their malformations. These include lymphedema, chylous ascites, coagulopathy, protein-losing enteropathy, and progressive bone loss (Lala et al., 2013; Croteau et al., 2014; Trenor and Chaudry, 2014; Andreoti et al., 2023). Patients with LMs in the thoracic cavity can also develop chylothorax, a complication that can cause respiratory distress, failure, and death (Lala et al., 2013; Ludwig et al., 2016; Andreoti et al., 2023). The overlapping symptoms, imaging features, and complications of CLAs make them challenging to diagnose, and their precise incidence is unknown (Trenor and Chaudry, 2014; Iacobas et al., 2020). However, the mortality rate of these diseases can be high, with the 5-year survival rate being as low as 51% (Croteau et al., 2014).

Next-generation sequencing studies have begun to shed light on the genetic basis of many CLAs. Somatic activating mutations in *PIK3CA*, *ARAF*, *BRAF*, *CBL*, *HRAS*, and *NRAS* have been found in sporadic CLAs (Manevitz-Mendelson et al., 2018; Barclay et al., 2019; Li et al., 2019; Ozeki et al., 2019; Rodriguez-Laguna et al., 2019; Foster et al., 2020; Grenier et al., 2023; Li et al., 2023). We and other groups recently identified somatic activating mutations in *KRAS* in CLA patients (Li et al., 2019; Nozawa et al., 2020; Homayun-Sepehr et al., 2021; Andreoti et al., 2023; Li et al., 2023; Sheppard et al., 2023). To date, five different activating *KRAS* mutations (p.G12V, p.G12D, p.G13D, p.Q61R, and p.A146T) have been identified in patients (Li et al., 2019; Nozawa et al., 2020; Homayun-Sepehr et al., 2021; Li et al., 2023; Sheppard et al., 2023). KRAS is a small GTPase that switches between inactive GDP-bound and active GTP-bound forms (Haigis, 2017). The *KRAS* mutations in CLA patients impair GTP hydrolysis, resulting in hyperactive downstream signaling (Haigis, 2017). To investigate the effect of hyperactive KRAS signaling on lymphatic vessels, we used the Cre-loxP system to conditionally express an active form of KRAS (Kras^G12D^) in lymphatic endothelial cells (LECs) during postnatal development (Homayun-Sepehr et al., 2021). We found that *Kras* ^*G12D*^-mutant mice developed intraosseous lymphatic vessels, a feature frequently seen in CLAs (Homayun-Sepehr et al., 2021). We also showed that the development of lymphatic valves and recruitment of lymphatic muscle cells were impaired in *Kras* ^*G12D*^-mutant mice (Homayun-Sepehr et al., 2021). Additionally, we found that MEK1/2 inhibition with trametinib suppressed the loss of lymphatic valves in newborn *Kras* ^*G12D*^-mutant mice (Homayun-Sepehr et al., 2021). Despite these recent advances, the mechanisms by which activating *KRAS* mutations cause CLAs remain incompletely understood. In the present study, we delineate the effect of hyperactive KRAS signaling on the development of the lymphatic system in mouse embryos. We also molecularly profile primary human LECs expressing an active form of KRAS and assess the effect of trametinib on KRAS mutant cells and embryos. Finally, we characterize *Kras* conditional knockout mice to determine the role of wild-type *Kras* in the formation and function of lymphatic vessels.

## Results

### *LEC* ^*KrasG12D*^ embryos exhibit multiple lymphatic vessel defects

**FIGURE 1:**
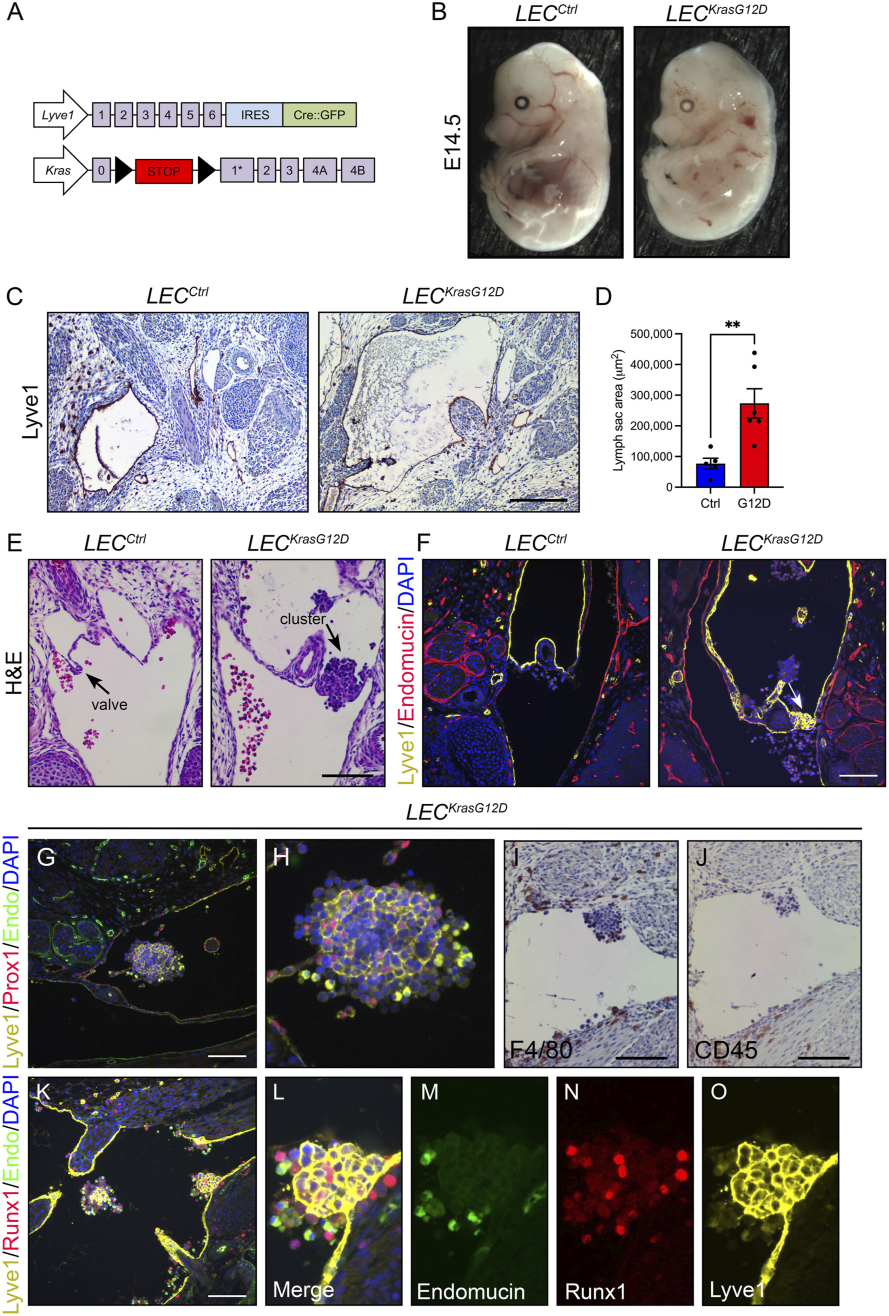
*LEC* ^*KrasG12D*^ embryos have enlarged jugular lymph sacs and malformed lymphovenous valves. **(A)**. Schematics of the *Lyve1-Cre* and *Kras* ^*LSL-G12D*^ alleles. **(B)**. Representative images of E14.5 *LEC* ^*Ctrl*^ and *LEC* ^*KrasG12D*^ embryos. The *LEC* ^*KrasG12D*^ embryo has edema. **(C)**. Transverse sections of E14.5 embryos stained with an anti-Lyve1 antibody (brown) and hematoxylin (purple). **(D)**. Jugular lymph sac area was significantly greater in *LEC* ^*KrasG12D*^ embryos (273391 ± 47694; *n* = 6 mice) than *LEC* ^*Ctrl*^ embryos (76949 ± 17566; *n* = 5 mice). **(E)**. Coronal sections of E14.5 embryos stained with hematoxylin and eosin (H&E). The arrow points to a lymphovenous valve in a *LEC* ^*Ctrl*^ embryo. The arrow points to a cluster of cells in the lymphovenous valve region in a *LEC* ^*KrasG12D*^ embryo. Six *LEC* ^*Ctrl*^ and five *LEC* ^*KrasG12D*^ embryos were analyzed. **(F)**. Coronal sections of E14.5 embryos stained with DAPI (blue) and antibodies against Lyve1 (yellow) and endomucin (red). The arrow points to a lymphovenous valve in a *LEC* ^*Ctrl*^ embryo. The arrow points to a cluster of Lyve1-positive cells in the lymphovenous valve region in a *LEC* ^*KrasG12D*^ embryo. Five *LEC* ^*Ctrl*^ and three *LEC* ^*KrasG12D*^ embryos were analyzed. **(G)**. Immunostaining revealed that cell clusters in the jugular lymph sacs of *LEC* ^*KrasG12D*^ embryos contained Prox1 and Lyve-1 double-positive LECs. Six *LEC* ^*KrasG12D*^ embryos were analyzed. **(H)**. Higher magnification image of cluster in panel **(G)**. (**I,J)**. Clusters also contained F4/80-positive cells **(I)** and CD45-positive cells **(J)**. **(K)**. Immunostaining revealed that clusters in *LEC* ^*KrasG12D*^ embryos contained Runx1-positive cells. (**L–O)**. Higher magnification images of the separate channels in panel **(K)**. Data are presented as mean ± SEM. **p < 0.01; unpaired Student’s *t*-tests. Scale bar in panel C = 200 µm. Scale bars in panels E, G, I, J, and K = 100 µm.

To characterize the effect of KRAS^G12D^ on lymphatic vessels during embryonic development, we bred *Lyve1-Cre* mice with *LSL-Kras* ^*G12D*^ mice to create control (*LEC* ^*Ctrl*^) and mutant (*LEC* ^*KrasG12D*^) embryos (Figure 1A). *Lyve1-Cre* mice exhibit Cre activity in LECs, macrophages, and blood endothelial cells in the yolk sac, liver, and lung and have been used by many groups to induce recombination in LECs (Pham et al., 2010; Dellinger et al., 2013; Cha et al., 2016; Cha et al., 2020; Geng et al., 2020). *LSL-Kras* ^*G12D*^ mice express oncogenic Kras (*KRAS* ^*G12D*^) from the endogenous *KRAS* locus following Cre-mediated recombination (Jackson et al., 2001). *LEC* ^*KrasG12D*^ mice exhibited profound edema at embryonic day 14.5 (E14.5). Edema was displayed by 0/16 *LEC* ^*Ctrl*^ embryos and 17/17 *LEC* ^*KrasG12D*^ embryos (*p* < 0.0001; Fisher’s exact test). Morphometric analysis of E14.5 embryos revealed that the jugular lymph sacs were significantly larger in *LEC* ^*KrasG12D*^ embryos than *LEC* ^*Ctrl*^ embryos (Figures 1C, D). Embryos with edema and enlarged jugular lymph sacs exhibit lymphovenous valve defects (Geng et al., 2016). Lymphovenous valves are located at the junction between the jugular lymph sacs and subclavian veins and prevent blood from entering lymphatic vessels (Geng et al., 2016). To determine whether *LEC* ^*KrasG12D*^ embryos display lymphovenous valve defects, we analyzed hematoxylin and eosin (H&E)-stained coronal sections of E14.5 embryos. *LEC* ^*Ctrl*^ embryos had lymphovenous valve leaflets that extended from the jugular lymph sacs into the veins (Figure 1E). In contrast, *LEC* ^*KrasG12D*^ embryos lacked well-defined valve leaflets at the lymphovenous junction (Figure 1E). Lymphovenous valves were observed in 6/6 *LEC* ^*Ctrl*^ embryos and 0/5 *LEC* ^*KrasG12D*^ embryos (*p* < 0.01 Fisher’s exact test). Immunofluorescence staining of coronal sections of E14.5 embryos for Lyve1 and endomucin confirmed that *LEC* ^*Ctrl*^ embryos, but not *LEC* ^*KrasG12D*^ embryos, had well-defined lymphovenous valves (Figure 1F). Instead of lymphovenous valves, *LEC* ^*KrasG12D*^ embryos frequently had cell clusters at lymphovenous junctions (Figure 1F). Cell clusters were also present in other regions in the jugular lymph sacs of *LEC* ^*KrasG12D*^ embryos. Cell clusters were observed in the jugular lymph sacs of 0/6 *LEC* ^*Ctrl*^ embryos and 8/10 *LEC* ^*KrasG12D*^ embryos (*p* < 0.01 Fisher’s exact test). Immunofluorescence staining revealed that the clusters contained numerous Lyve1 and Prox1 double-positive LECs (Figures 1G, H). It was recently reported that mouse embryos harboring mutations in an enhancer element located 11 kb upstream of *Prox1* (*Prox1-11 kb* ^*Δ/Δ*^) exhibit similar cell clusters in their jugular lymph sacs (Kazenwadel et al., 2023). Immunofluorescence staining with a panel of markers revealed that the clusters in *Prox1-11 kb* ^*Δ/Δ*^ embryos contained Prox1-positive cells and cells expressing various hematopoietic markers (Kazenwadel et al., 2023). To further characterize the cellular landscape of clusters in the jugular lymph sacs of *LEC* ^*KrasG12D*^ embryos, we stained sections of E14.5 embryos with a panel of antibodies. This revealed that clusters in *LEC* ^*KrasG12D*^ embryos contained heterogeneous populations of CD45, F4/80, and RUNX1-positive cells (Figures I–O). Therefore, the cell clusters in the jugular lymph sacs of *LEC* ^*KrasG12D*^ embryos include LECs and various hematopoietic cells.

**FIGURE 2:**
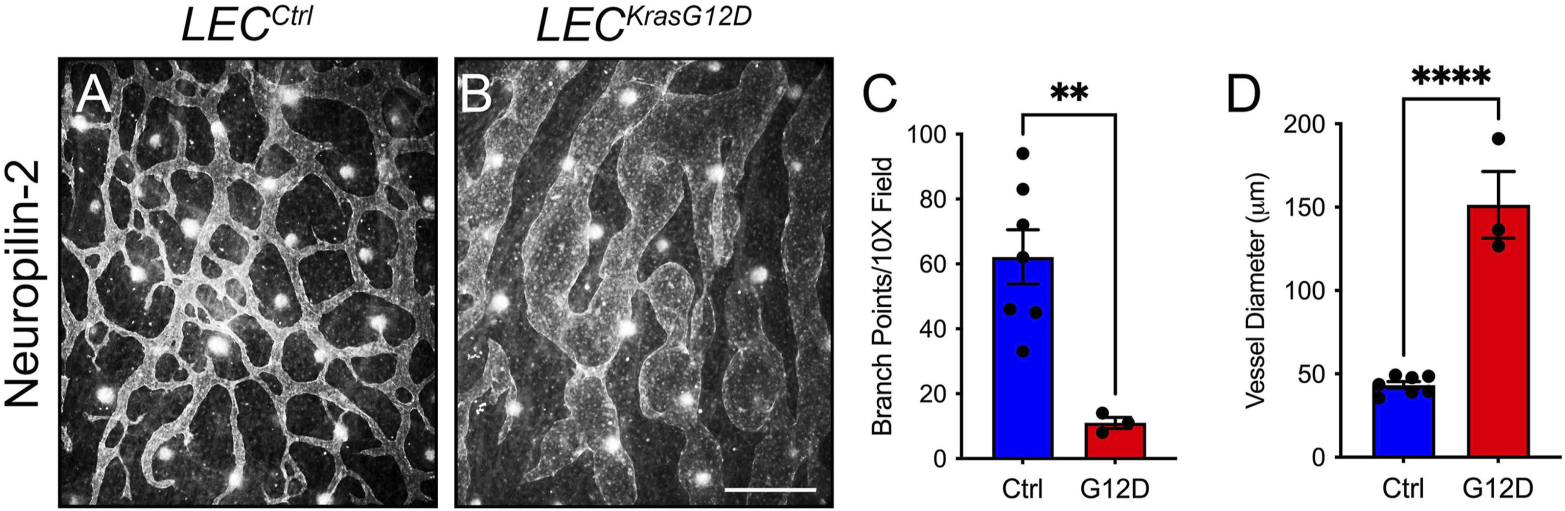
*LEC* ^*KrasG12D*^ embryos have abnormal dermal lymphatic vessels. **(A,B)**. Back skin whole-mounts from E15.5 embryos stained for neuropilin-2. **(C)**. *LEC* ^*KrasG12D*^ embryos (11.00 ± 1.732; *n* = 3 mice) had significantly fewer lymphatic vessel branch points than *LEC* ^*Ctrl*^ embryos (62.14 ± 8.382; *n* = 7 mice). **(D)**. Lymphatic vessel diameter was significantly greater in *LEC* ^*KrasG12D*^ embryos (151.4 ± 20.05; *n* = 3 mice) than in *LEC* ^*Ctrl*^ embryos (43.30 ± 2.047; *n* = 7 mice). Data are presented as mean ± SEM. ** *p* < 0.01, **** *p* < 0.0001; unpaired Student’s *t*-tests. Scale bar = 250 µm.

We next characterized the effect of KRAS^G12D^ expression on the patterning of lymphatic vessels. Whole-mount immunofluorescence staining of E15.5 back skin for neuropilin-2 revealed that the dermal lymphatic network in *LEC* ^*KrasG12D*^ embryos was irregular and less complex than the network in *LEC* ^*Ctrl*^ embryos (Figures 2A, B). *LEC* ^*KrasG12D*^ embryos had significantly fewer lymphatic vessel branch points than *LEC* ^*Ctrl*^ embryos (Figure 2C). Additionally, the diameter of lymphatic vessels was significantly greater in *LEC* ^*KrasG12D*^ embryos than in *LEC* ^*Ctrl*^ embryos (Figure 2D).

### Hyperactive KRAS signaling in LECs induces changes in cell shape, proliferation, and migration

**FIGURE 3:**
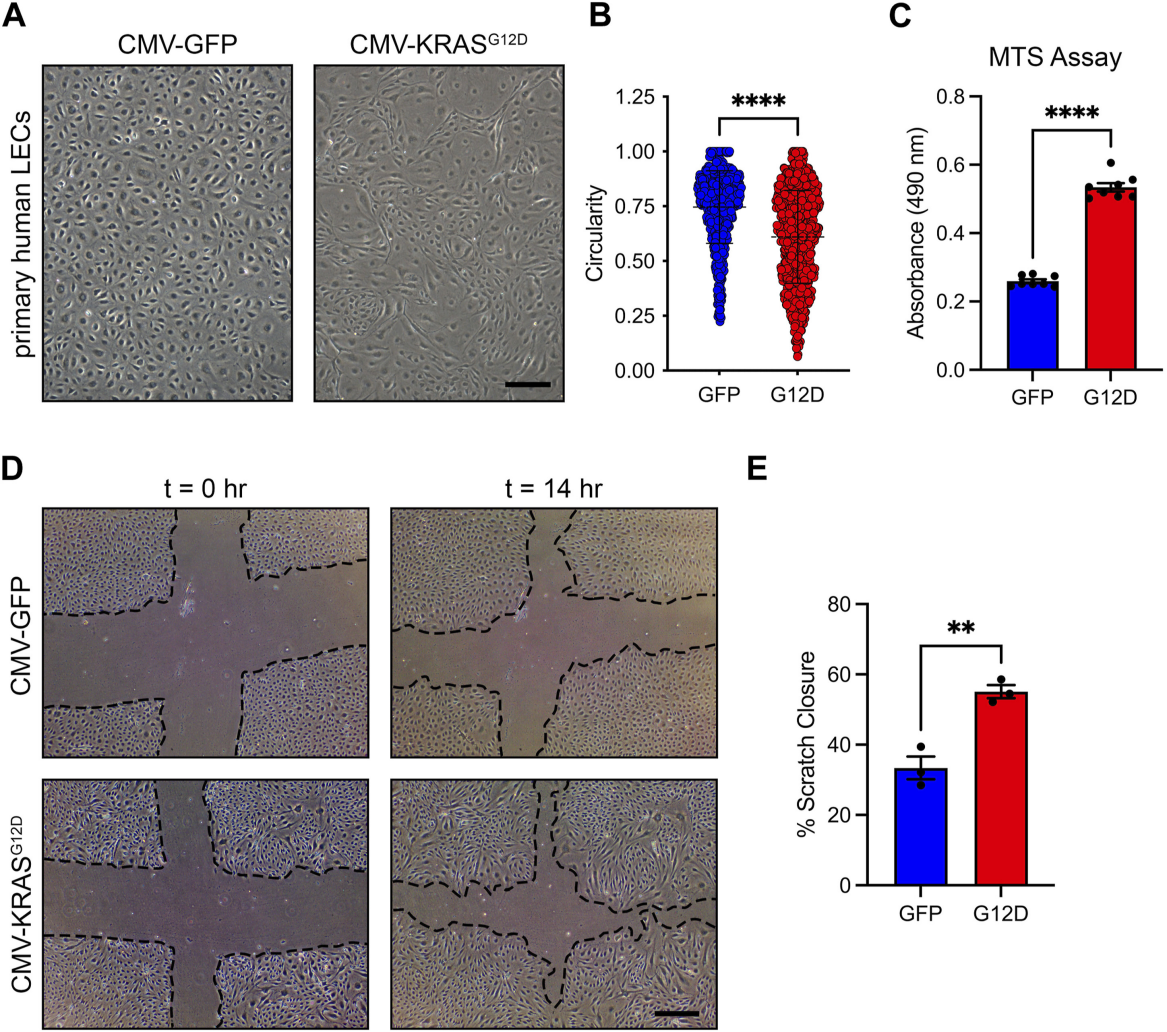
KRAS^G12D^ induces cell morphological changes, proliferation, and migration. **(A)**. Brightfield images of GFP-LECs and KRAS^G12D^-LECs. The images were taken 72 h after treating primary human LECs with lentivirus particles that express GFP or KRAS^G12D^. GFP-LECs exhibit a normal cobblestone morphology, whereas KRAS^G12D^-LECs exhibit a spindle morphology. **(B)**. Circularity index measurements for GFP-LECs and KRAS^G12D^-LECs. **(C)**. MTS viability assay results for GFP-LECs and KRAS^G12D^-LECs. Viability was measured 72 h after treating cells with lentivirus particles that express GFP or KRAS^G12D^. **(D)**. Representative images of GFP-LECs and KRAS^G12D^-LECs taken 0 and 14 h after scratching confluent monolayers of cells. **(E)**. Graph showing scratch closure area 14 h after wounding. KRAS^G12D^-LECs closed the scratched area significantly faster than GFP-LECs. Data are presented as mean ± SEM. **p < 0.01, ****p < 0.0001; unpaired Student’s *t*-tests. Scale bars = 300 µm.

To investigate the molecular mechanisms by which hyperactive KRAS signaling causes the maldevelopment of lymphatic vessels, we used lentiviral vectors to transiently express GFP (vector control) or KRAS^G12D^ in primary human LECs. We based our approach on previously published studies that modeled KRAS-induced arteriovenous malformations (AVMs) by treating cells with a control vector or a vector expressing mutant KRAS (Nikolaev et al., 2018; Fish et al., 2020). We found that KRAS^WT^-lentivirus had a similar effect on cell signaling in LECs across a range of MOIs as GFP-lentivirus (Supplementary Figure S1). Therefore, we believe that the GFP-lentivirus is a suitable control for our experiments. We found that GFP-LECs retained a standard cobblestone-like shape, whereas KRAS^G12D^-LECs exhibited an elongated/spindled shape (Figure 3A). Circularity index measurements revealed that KRAS^G12D^-LECs were significantly less circular than GFP-LECs (Figure 3B). We next assessed the effect of KRAS^G12D^ on cellular processes associated with the growth of lymphatic vessels. MTS assays demonstrated that KRAS^G12D^ stimulated LEC proliferation, and scratch assays showed that it promoted LEC migration (Figures 3C–E). These data indicate that hyperactive KRAS signaling in LECs promotes cellular behaviors associated with the growth of lymphatic vessels.

### KRAS^G12D^ induces PI3K/AKT and MAPK signaling and changes in gene expression in LECs

**FIGURE 4:**
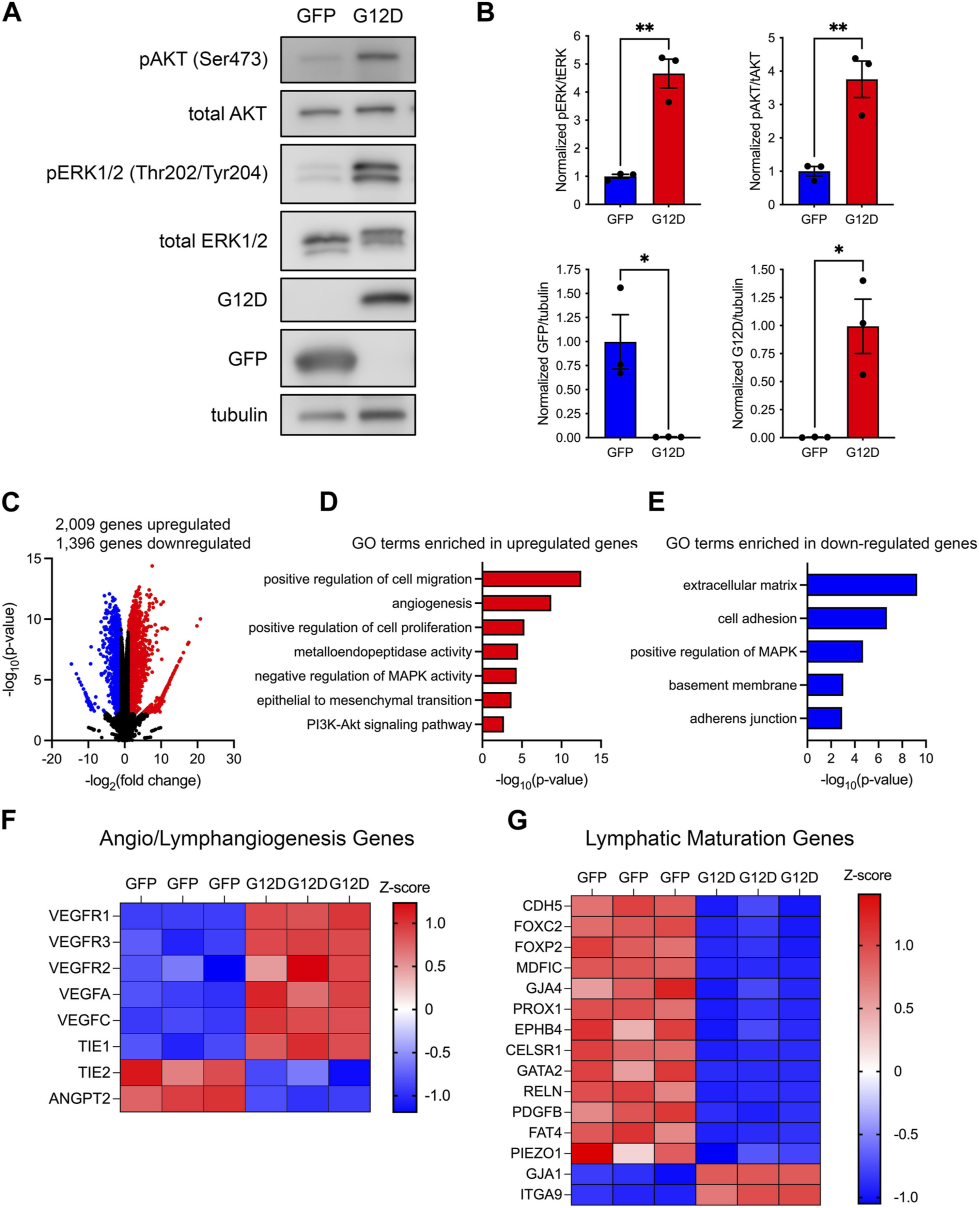
KRAS^G12D^ expression in LECs induces PI3K and MAPK signaling and changes in gene expression. **(A)**. Western blot results for phospho-AKT, AKT, phospho-ERK1/2, ERK1/2, GFP, KRAS^G12D^ (mutation-specific antibody), and tubulin. Protein lysates were made 72 h after treating primary human LECs with GFP or KRAS^G12D^ expressing lentivirus particles. **(B)**. Graphs of Western blot results. Phospho-AKT and phospho-ERK1/2 levels were significantly higher in KRAS^G12D^-LECs than GFP-LECs. GFP-LECs specifically expressed GFP, and KRAS^G12D^-LECs specifically expressed KRAS^G12D^. **(C)**. Volcano plot of RNA-Seq results comparing KRAS^G12D^-LECs to GFP-LECs. Two thousand and nine genes were upregulated (red dots), and 1,396 genes were downregulated (blue dots) by KRAS^G12D^ (log_2_ fold-change ≥1 or ≤ −1; FDR <0.01). **D,E.** Select GO terms associated with genes upregulated by KRAS^G12D^ **(D)** and genes downregulated by KRAS^G12D^ **(E)**. **(F,G)**. Heatmaps of genes that regulate angio/lymphangiogenesis **(F)** and genes that control lymphatic valve development or lymphatic muscle cell recruitment **(G)**. Data are presented as mean ± SEM. * *p* < 0.05, ** *p* < 0.01; unpaired Student’s *t*-tests.

KRAS^G12D^ influences cell survival, proliferation, and migration by activating a diverse set of downstream signal transduction pathways. Two prominent pathways activated by KRAS^G12D^ are the phosphatidylinositol 3-kinase (PI3K) and mitogen-activated protein kinase (MAPK) pathways. To determine whether KRAS^G12D^ activated these pathways in LECs, we assessed the phosphorylation of AKT and ERK1/2 in GFP-LECs and KRAS^G12D^-LECs by immunoblotting. This revealed that the levels of phospho-AKT and phopsho-ERK1/2 were significantly higher in KRAS^G12D^-LECs than GFP-LECs (Figures 4A, B). Immunoblotting also demonstrated that GFP-LECs specifically expressed GFP and that KRAS^G12D^-LECs specifically expressed KRAS^G12D^ (Figures 4A, B).

To determine the impact of KRAS^G12D^-induced alterations in cell signaling on gene expression, we analyzed the transcriptomes of GFP-LECs and KRAS^G12D^-LECs by bulk RNA-sequencing. We found that 2,009 genes were upregulated, and 1,396 genes were downregulated in KRAS^G12D^-LECs compared to GFP-LECs (Figure 4C). Analysis of gene ontology (GO) terms showed that genes associated with angiogenesis, cell proliferation, cell migration, metalloendopeptidase activity, and epithelial-to-mesenchymal transition were upregulated by KRAS^G12D^ (Figure 4D). The RAS/MAPK pathway is regulated by a series of negative feedback mechanisms (Lake et al., 2016). These negative feedback mechanisms include rapid responses mediated by protein kinases and transcriptionally induced mechanisms. Active RAS/MAPK signaling in cancer cells induces the expression of negative regulators of the RAS/MAPK pathway, such as MAPK phosphatases (e.g., DUSP4) and Sprouty (Spry) proteins (Lake et al., 2016). We found that KRAS^G12D^ expression in LECs also induces the expression of negative regulators of the RAS/MAPK pathway (Figure 4D). GO term analysis also demonstrated that genes associated with cell adhesion, adherens junctions, and positive regulation of MAPK activity were downregulated by KRAS^G12D^ (Figure 4E). We then looked closer at the expression of genes that promote angio/lymphangiogenesis and genes that regulate the maturation of lymphatic vessels (valve development and recruitment of lymphatic muscle cells). We found that the expression of several angio/lymphangiogenic genes was increased, whereas the expression of lymphatic maturation genes was decreased in KRAS^G12D^-LECs compared to GFP-LECs (Figures 4F, G).

The growth factor VEGF-C stimulates lymphangiogenesis by activating the receptor tyrosine kinase VEGFR3. VEGF-C/VEGFR3 signaling was recently shown to promote the progression of PIK3CA-induced lymphatic malformations (Martinez-Corral et al., 2020). Because VEGF-C and VEGFR3 transcripts were elevated in KRAS^G12D^-LECs, we further characterized VEGF-C/VEGFR3 signaling in KRAS^G12D^-LECs. We found by ELISA that KRAS^G12D^-LECs expressed and secreted more VEGF-C than GFP-LECs (Supplementary Figure S2). We also found that phospho-VEGFR3 levels were greater in KRAS^G12D^-LECs than in GFP-LECs (Supplementary Figure S2). Although phospho-VEGFR3 levels were high in KRAS^G12D^-LECs, they could be driven even higher by the addition of recombinant VEGF-C. Additionally, phospho-AKT, but not phospho-ERK1/2 levels, increased in KRAS^G12D^-LECs following stimulation with recombinant VEGF-C (Supplementary Figure S2). These data suggest that KRAS^G12D^-LECs, like PIK3CA^H1047R^-LECs (Martinez-Corral et al., 2020), are responsive to exogenous VEGF-C.

Changes in global gene expression are mediated, in part, by the activity of transcription factors. We analyzed our bulk transcriptome data using Metascape (Zhou et al., 2019) to identify transcription regulatory networks altered by hyperactive KRAS signaling. We found that genes regulated by the transcription factors JUN, FOS, STAT3, ETS1, and ETS2 were upregulated by KRAS^G12D^, whereas genes regulated by TP53, E2F1, and HIF1α were downregulated by KRAS^G12D^ (Supplementary Figure S3).

### Trametinib increases the circularity of KRAS^G12D^-LECs and suppresses the proliferation and migration of KRAS^G12D^-LECs

**FIGURE 5:**
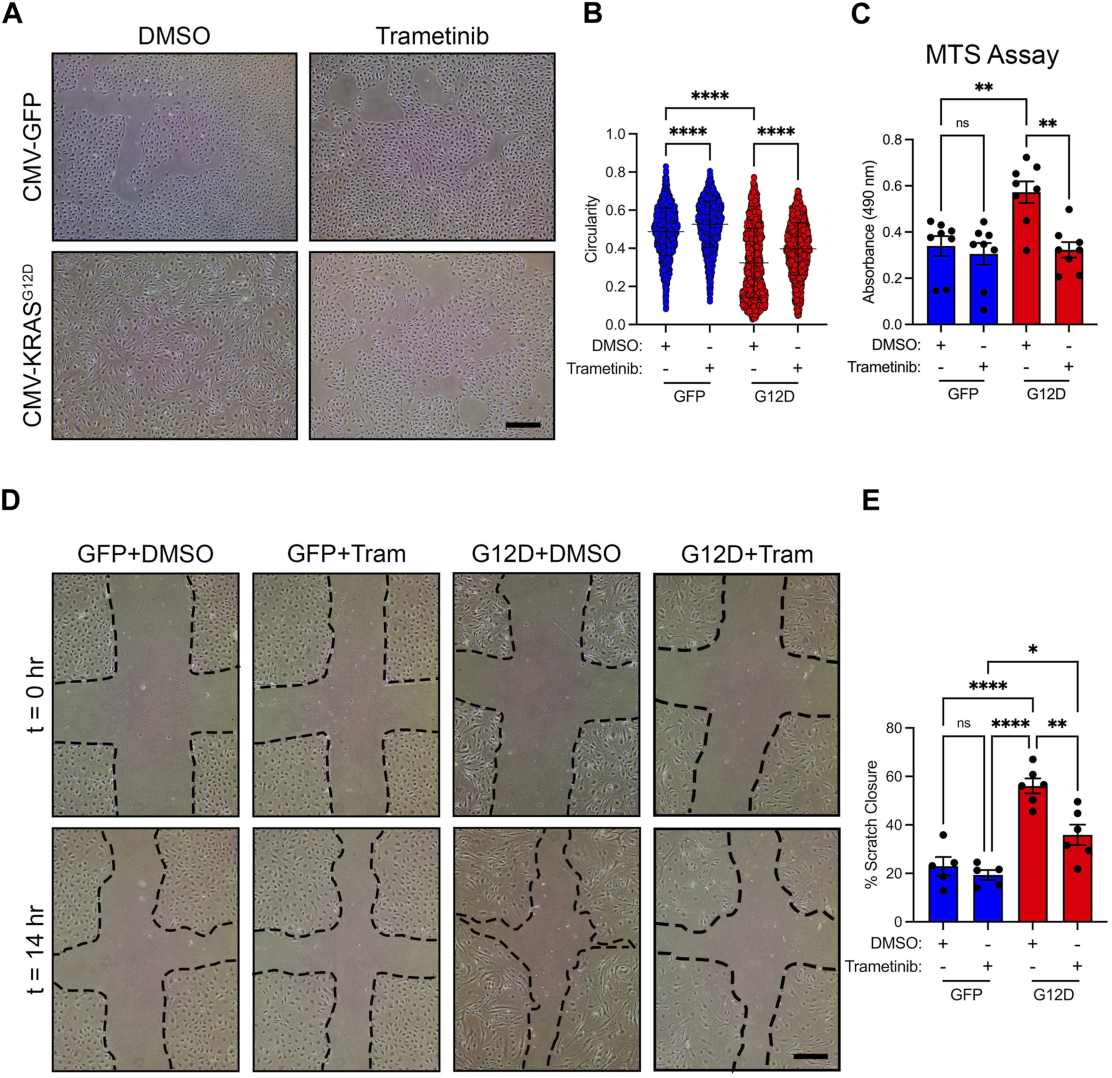
Trametinib decreases KRAS^G12D^-induced cell shape changes, proliferation, and migration. **(A)**. Representative brightfield images of GFP-LECs and KRAS^G12D^-LECs treated with DMSO or trametinib (10 nM) for 48 h. **(B)**. Circularity index measurements for GFP-LECs and KRAS^G12D^-LECs treated with DMSO or trametinib (10 nM). Trametinib significantly increased the circularity of KRAS^G12D^-LECs. **(C)**. MTS viability assay results for GFP-LECs and KRAS^G12D^-LECs treated with DMSO or trametinib (10 nM) for 72 h. Trametinib decreased the proliferation of KRAS^G12D^-LECs. **(D)**. Representative images of GFP-LECs and KRAS^G12D^-LECs taken 0 or 14 h after scratching confluent monolayers of cells. Cells were treated with DMSO or trametinib (10 nM) immediately after scratching. **(E)**. Graph showing scratch closure area 14 h after wounding. Trametinib-treated KRAS^G12D^-LECs closed the scratched area significantly slower than DMSO-treated KRAS^G12D^-LECs. Data are presented as mean ± SEM. * *p* < 0.05, ** *p* < 0.01, **** *p* < 0.0001, ns = not significant; ANOVA Tukey’s multiple comparisons test. Scale bars = 300 µm.

Trametinib (an FDA-approved MEK1/2 inhibitor) is an emerging treatment for lymphatic anomalies. Trametinib improves patient symptoms (e.g., pulmonary function and pleural effusions/chylothorax) and alters lymph drainage pathways in patients (Li et al., 2019; Foster et al., 2020; Chowers et al., 2022). We previously showed that trametinib attenuates KRAS^G12D^-induced lymphatic valve loss in neonatal mice (Homayun-Sepehr et al., 2021). To investigate the effect of trametinib on cellular processes associated with lymphangiogenesis, we treated GFP-LECs and KRAS^G12D^-LECs with DMSO (vehicle) or trametinib. We found that trametinib-treated KRAS^G12D^-LECs had more of a standard cobblestone appearance than DMSO-treated KRAS^G12D^-LECs (Figure 5A). Circularity index measurements revealed that trametinib-treated KRAS^G12D^-LECs were significantly more circular than DMSO-treated KRAS^G12D^-LECs (Figure 5B). Trametinib also decreased the proliferation and migration of KRAS^G12D^-LECs (Figures 5C–E). These data suggest that MEK1/2 inhibition normalizes aberrant cell behaviors caused by hyperactive KRAS signaling in LECs.

**FIGURE 6:**
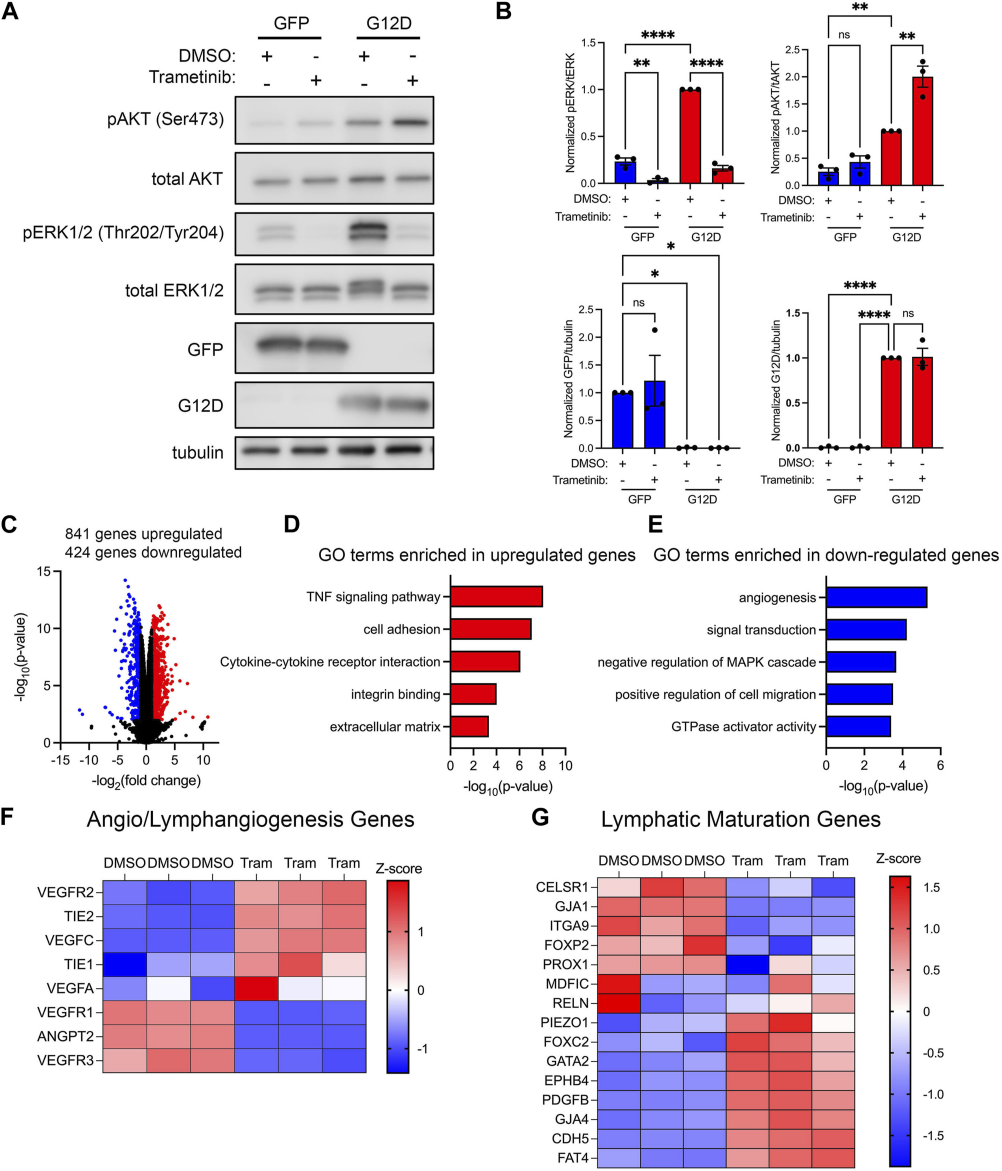
Trametinib decreases MAPK signaling and increases the expression of lymphatic maturation genes. **(A)**. Western blot analysis of phospho-AKT, AKT, phospho-ERK1/2, ERK1/2, GFP, KRAS^G12D^ (mutation-specific antibody), and tubulin. Protein lysates were made 16 h after treating GFP-LECs and KRAS^G12D^-LECs with DMSO or trametinib (10 nM). **(B)**. Graphs of Western blot results. Trametinib increased phospho-AKT levels and decreased phospho-ERK1/2 levels in KRAS^G12D^-LECs. **(C)**. Volcano plot of RNA-Seq data comparing trametinib-treated KRAS^G12D^-LECs to DMSO-treated KRAS^G12D^-LECs. RNA was isolated 16 h after treating cells with DMSO or trametinib (10 nM). Eight hundred forty-one genes were upregulated (red dots), and 424 genes were downregulated (blue dots) by trametinib (log_2_ fold-change ≥1 or ≤ −1; FDR <0.02). **(D,E)**. Select GO terms associated with genes upregulated by trametinib **(D)** and genes downregulated by trametinib **(E)**. **(F,G)**. Heatmaps of genes that regulate angio/lymphangiogenesis **(F)** and genes that control lymphatic valve development or lymphatic muscle cell recruitment **(G)**. Data are presented as mean ± SEM. * *p* < 0.05, ** *p* < 0.01, **** *p* < 0.0001, ns = not significant; ANOVA Tukey’s multiple comparisons test and Dunnett’s multiple comparisons test.

To characterize the effect of trametinib on cell signaling, we treated GFP-LECs and KRAS^G12D^-LECs with DMSO or trametinib, and then assessed the phosphorylation of AKT and ERK1/2 by immunoblotting. We found that trametinib significantly decreased ERK1/2 phosphorylation in GFP-LECs and KRAS^G12D^-LECs (Figures 6A, B). Interestingly, trametinib modestly increased AKT phosphorylation in GFP-LECs and significantly increased AKT phosphorylation in KRAS^G12D^-LECs (Figures 6A, B). Trametinib and other MEK1/2 inhibitors have also been shown to induce AKT phosphorylation in a variety of cancer cell lines (Chen et al., 2017; Tsubaki et al., 2019). Although trametinib affected ERK1/2 and AKT phosphorylation, it did not affect the expression of GFP in GFP-LECs or KRAS^G12D^ in KRAS^G12D^-LECs (Figures 6A, B).

To determine the impact of trametinib on gene expression, we analyzed the transcriptomes of DMSO and trametinib-treated KRAS^G12D^-LECs by bulk RNA sequencing. We found that 841 genes were upregulated, and 424 genes were downregulated in trametinib-treated KRAS^G12D^-LECs compared to DMSO-treated KRAS^G12D^-LECs (Figure 6C). GO term analysis showed that genes associated with TNF signaling, cell adhesion, integrin binding, and cytokine-cytokine receptor signaling were upregulated by trametinib (Figure 6D). Additional GO term analysis demonstrated that genes associated with angiogenesis, GTPase activity, and cell migration were downregulated by trametinib (Figure 6E). Trametinib also decreased the expression of negative regulators of the MAPK pathway (Figure 6E). We then examined the expression of genes that promote angio/lymphangiogenesis and genes that regulate the maturation of lymphatic vessels. We found that trametinib decreased the expression of several angio/lymphangiogenic genes, and increased the expression of several genes that promote the maturation of lymphatic vessels (Figures 6F, G).

To determine the effect of trametinib on transcription regulatory networks in KRAS^G12D^-LECs, we again analyzed our bulk transcriptome data using Metascape. We found that genes regulated by the transcription factors TP53, NFKB1, and IRF-1 were upregulated by trametinib, whereas genes regulated by CREB, JUN, FOS, ETS1, and ETS2 were downregulated by trametinib (Supplementary Figure S3).

### Trametinib suppresses KRAS^G12D^-induced lymphatic vessel hyperplasia

**FIGURE 7:**
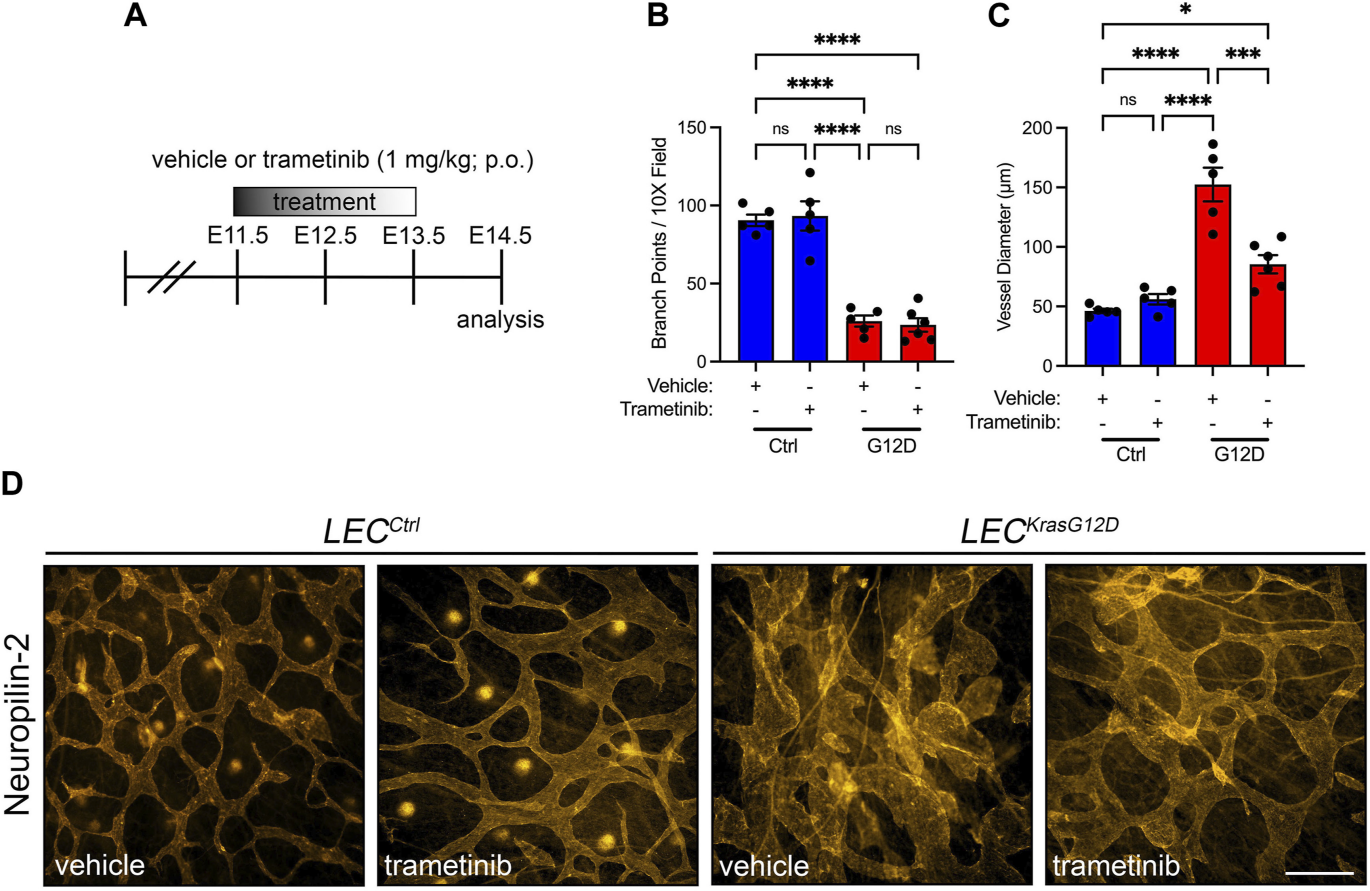
Trametinib partially suppresses KrasG12D-induced enlargement of lymphatic vessels. **(A)**. Schematic showing when mice received vehicle or trametinib (1 mg/mL; p.o.; q.d.). **(B)**. Lymphatic vessel branch points for vehicle-treated *LEC* ^*Ctrl*^ (90.50 ± 3.647; *n* = 5 mice), trametinib-treated *LEC* ^*Ctrl*^ (93.30 ± 9.332; *n* = 5 mice), vehicle-treated *LEC* ^*KrasG12D*^ (25.97 ± 3.578; *n* = 5 mice), and trametinib-treated *LEC* ^*KrasG12D*^ mice (23.50 ± 4.363; *n* = 6 mice). **(C)**. Lymphatic vessel diameter measurements for vehicle-treated *LEC* ^*Ctrl*^ (46.24 ± 1.872; *n* = 5 mice), trametinib-treated *LEC* ^*Ctrl*^ (56.02 ± 4.381; *n* = 5 mice), vehicle-treated *LEC* ^*KrasG12D*^ (152.4 ± 14.14; *n* = 5 mice), and trametinib-treated *LEC* ^*KrasG12D*^ mice (85.42 ± 7.590; *n* = 6 mice). **(D)**. Back skin whole-mounts from E14.5 embryos stained for neuropilin-2. Data are presented as mean ± SEM. * *p* < 0.05, *** *p* < 0.001, **** *p* < 0.0001, ns = not significant; ANOVA Tukey’s multiple comparisons test. Scale bar = 250 µm.

We previously showed that trametinib suppresses the loss of lymphatic valves in newborn mice that express Kras^G12D^ in their LECs. We did not assess the effect of trametinib on the diameter of lymphatic vessels. Evaluating the impact of trametinib on the size of lymphatic vessels is clinically relevant because patients with RAS pathway mutations have dilated lymphatic channels (Liu et al., 2022). To assess the effect of trametinib on lymphatic hyperplasia in *LEC* ^*KrasG12D*^ embryos, we treated embryos with vehicle or trametinib from E11.5 to E13.5 and stained back skin from E14.5 embryos with an antibody against neuropilin-2 (Figures 7A–D; Supplementary Figure S4). We found that trametinib did not significantly affect the number of lymphatic branch points in *LEC* ^*KrasG12D*^ embryos (Figure 7B). However, trametinib significantly decreased the diameter of lymphatic vessels in *LEC* ^*KrasG12D*^ embryos (Figure 7C). These data suggest that pharmacologic inhibition of MAPK signaling can partially suppress Kras^G12D^-induced lymphatic hyperplasia *in vivo*.

### Genetic inhibition of MAPK signaling in LECs partially suppresses KRAS^G12D^-induced lymphatic vessel hyperplasia

**FIGURE 8:**
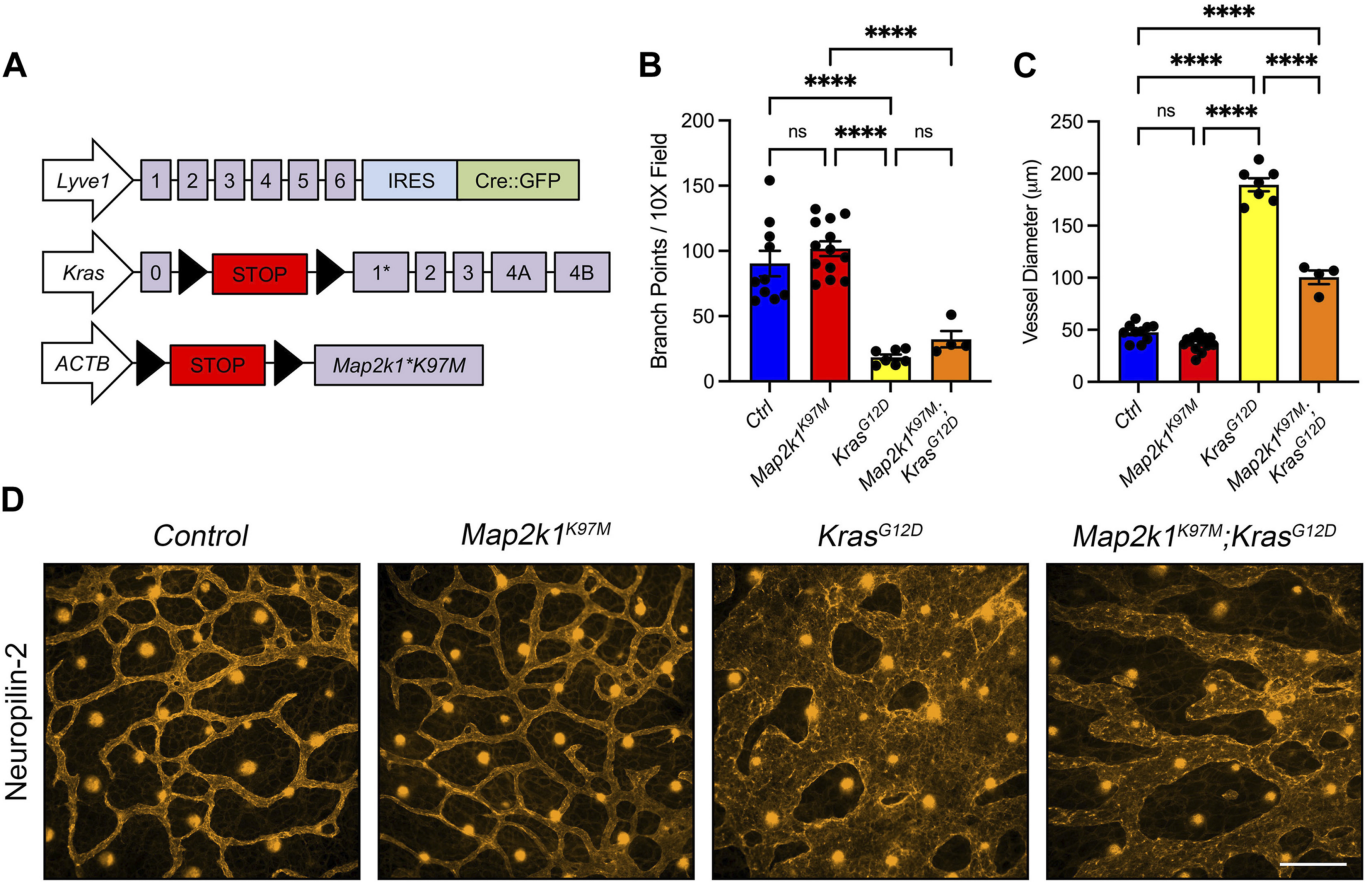
A dominant-negative form of MEK1 (Map2k1^K97M^) partially suppresses Kras^G12D^-induced enlargement of lymphatic vessels. **(A)**. Schematics of the *Lyve1-Cre*, *Kras* ^*LSL-G12D*^, and *Tg* ^*LSL-Map2k1K97M*^ alleles. **(B)**. Lymphatic vessel branch points for *LEC* ^*Ctrl*^ (90.38 ± 9.783; *n* = 10 mice), *LEC* ^*Map2k1K97M*^ (101.8 ± 5.693; *n* = 13 mice), *LEC* ^*KrasG12D*^ (18.38 ± 2.151; *n* = 7 mice), and *LEC* ^*Map2k1K97M;KrasG12D*^ (32.33 ± 6.320; *n* = 4 mice). **(C)**. Lymphatic vessel diameter measurements for *LEC* ^*Ctrl*^ (47.54 ± 2.628; *n* = 10 mice), *LEC* ^*Map2k1K97M*^ (37.05 ± 1.972; *n* = 13 mice), *LEC* ^*KrasG12D*^ (189.2 ± 6.192; *n* = 7 mice), and *LEC* ^*Map2k1K97M;KrasG12D*^ (100.4 ± 6.485; *n* = 4 mice). The *Tg* ^*LSL-Map2k1K97M*^ allele significantly decreased lymphatic vessel diameter in *LEC* ^*KrasG12D*^ embryos. **(D)**. Back skin whole-mounts from E15.5 embryos stained for neuropilin-2. Data are presented as mean ± SEM. **** *p* < 0.0001, ns = not significant; ANOVA Tukey’s multiple comparisons test. Scale bar = 250 µm.

Trametinib blocks MEK1/2 activity throughout the body. To determine whether specifically blocking MAPK activation in LECs could suppress KRAS^G12D^-induced lymphatic vessel hyperplasia, we obtained *LSL-Map2k1* ^*K97M*^ transgenic mice. *LSL-Map2k1* ^*K97M*^ transgenic mice express a dominant-negative form of human MEK1 following Cre-mediated removal of an upstream transcriptional stop sequence (Figure 8A) (Kelleher et al., 2004). The K97M mutation abolishes MEK1’s kinase activity but does not affect its ability to interact with ERK1 and ERK2 (Kelleher et al., 2004). To assess the effect of the Map2k1^K97M^ mutation on lymphatic vessel development, we collected back skin from E15.5 embryos and stained it for neuropilin-2 (Figures 8B–D; Supplementary Figure S5). We found that the number of lymphatic vessel branch points was not significantly different between *LEC* ^*Ctrl*^ and *LEC* ^*Map2k1K97M*^ embryos. The number of branch points was also not significantly different between *LEC* ^*KrasG12D*^ and *LEC* ^*KrasG12D;Map2k1K97M*^ embryos (Figure 8B). The diameter of lymphatic vessels was slightly lower in *LEC* ^*Map2k1K97M*^ embryos compared to *LEC* ^*Ctrl*^ embryos. However, this did not reach statistical significance. In contrast, lymphatic vessel diameter was significantly smaller in *LEC* ^*KrasG12D;Map2k1K97M*^ embryos compared to *LEC* ^*KrasG12D*^ embryos (Figure 8C). These results suggest that blocking MAPK activation in LECs partially suppresses KRAS^G12D^-induced lymphatic vessel hyperplasia.

### Conditional knockout of *Kras* in LECs does not impair the development or function of lymphatic vessels

**FIGURE 9:**
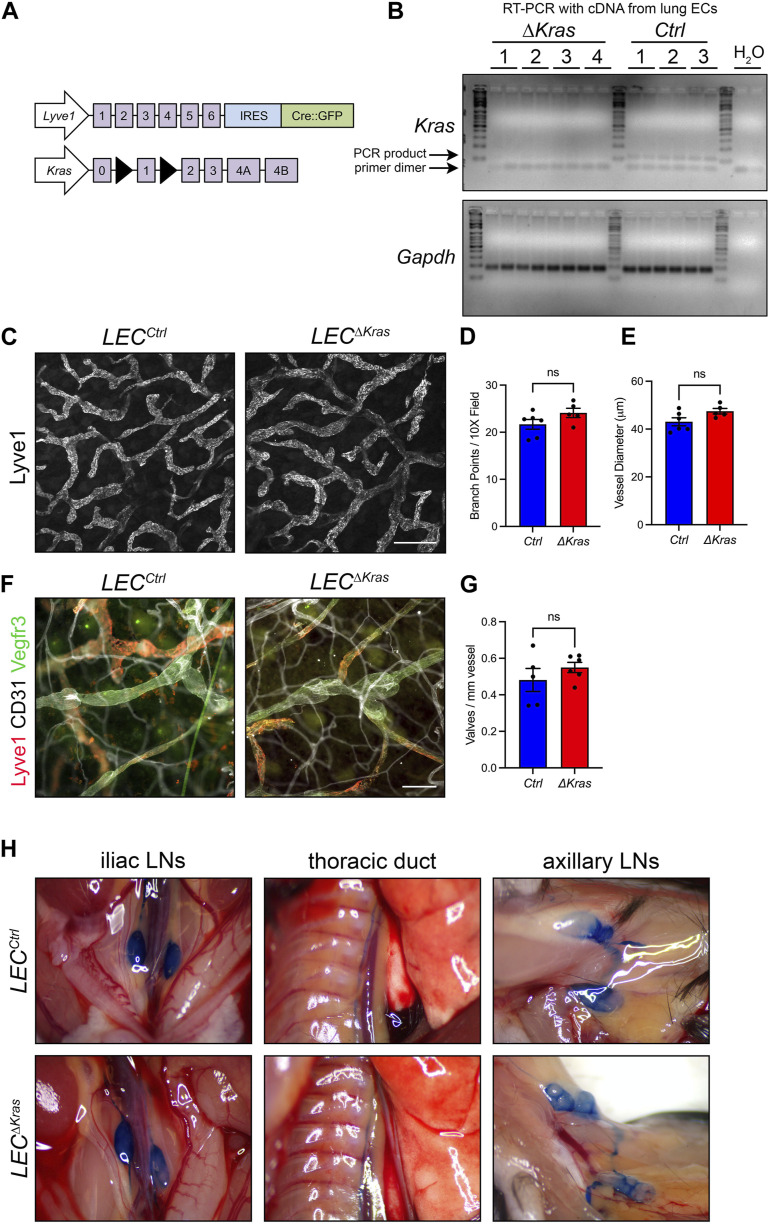
Conditional knockout of *Kras* in LECs does not impair the development or function of lymphatic vessels. **(A)**. Schematics of the *Lyve1-Cre* and *Kras* ^*loxp*^ alleles. **(B)**. RT-PCR results for *Kras* and *Gapdh* using cDNA generated from RNA isolated from pulmonary endothelial cells from *LEC* ^*Ctrl*^ and *LEC* ^*ΔKras*^ mice. **(C)**. Ear skin whole-mount preparations from *LEC* ^*Ctrl*^ and *LEC* ^*ΔKras*^ mice stained for Lyve1. **(D)**. The number of lymphatic vessel branch points was not significantly different between *LEC* ^*Ctrl*^ (21.68 ± 1.049; *n* = 6 mice) and *LEC* ^*ΔKras*^ mice (24.10 ± 0.9766; *n* = 5 mice). **(E)**. The diameter of lymphatic vessels was not significantly different between *LEC* ^*Ctrl*^ (43.05 ± 1.669; *n* = 6 mice) and *LEC* ^*ΔKras*^ mice (47.47 ± 1.141; *n* = 5 mice). **(F)**. Ear skin whole-mount preparations from *LEC* ^*Ctrl*^ and *LEC* ^*ΔKras*^ mice stained for Lyve1, CD31, and Vegfr3. **(G)**. The number of lymphatic valves/mm vessel was not significantly different between *LEC* ^*Ctrl*^ (0.4812 ± 0.06282; *n* = 5 mice) and *LEC* ^*ΔKras*^ mice (0.5495 ± 0.02791; *n* = 6 mice). **(H)**. Intradermally injected Evans blue dye was effectively transported from injection sites in *LEC* ^*Ctrl*^ (*n* = 4 mice) and *LEC* ^*ΔKras*^ mice (*n* = 4 mice). Data are presented as mean ± SEM. ns = not significant; unpaired Student’s *t*-tests. Scale bar in panel C = 250 µm. Scale bar in panel F = 100 µm.

Through gain-of-function experiments, we found that mutant KRAS causes lymphatic vessel hyperplasia and defects in the maturation of lymphatic vessels. However, little is known about the role of wild-type KRAS in the development of the lymphatic system. This is partly because global *Kras* knockout mice die during embryonic development before the lymphatic network fully forms (Johnson et al., 1997; Koera et al., 1997). Therefore, we set out to conditionally knockout *Kras* in LECs to identify its function in the development of lymphatic vessels. To conditionally knockout *Kras* in LECs, we bred *Lyve1-Cre* mice with *Kras* ^*loxp*^ mice to generate control (*LEC* ^*Ctrl*^) and *Lyve1-Cre;Kras* ^*loxp/loxp*^ (*LEC* ^*ΔKras*^) mice (Figure 9A). We found that *LEC* ^*ΔKras*^ mice were viable and born at their expected Mendelian frequency. To assess the efficiency of *Kras* deletion in *LEC* ^*ΔKras*^ mice, we first isolated RNA from CD31-positive cells collected from the lungs of 3 to 5-week-old *LEC* ^*Ctrl*^ and *LEC* ^*ΔKras*^ mice. We then performed reverse transcription (RT)-PCR for *Kras* and *Gapdh* (Figure 9B). This experiment demonstrated that *Kras* was efficiently deleted in *LEC* ^*ΔKras*^ mice. To determine whether *LEC* ^*ΔKras*^ mice have abnormal lymphatic vessels, we stained ear skin from 3 to 5-week-old mice with an antibody against Lyve1 (Figure 9C). We found that the number of lymphatic branch points and the diameter of lymphatic vessels were not significantly different between *LEC* ^*Ctrl*^ and *LEC* ^*ΔKras*^ mice (Figures 9D, E). We also stained ears for Lyve1, CD31, and VEGFR3 to identify lymphatic valves. We counted the number of CD31-positive valves in VEGFR3-positive-Lyve1-negative vessels and found that the number of lymphatic valves was not significantly different between *LEC* ^*Ctrl*^ and *LEC* ^*ΔKras*^ mice (Figures 9F,G). To assess lymphatic function in *LEC* ^*ΔKras*^ mice, we injected the hind paws and forepaws of mice with Evans blue dye (EBD). EBD was effectively transported from the hind paws to the iliac lymph nodes, then to the thoracic duct in *LEC* ^*Ctrl*^ and *LEC* ^*ΔKras*^ mice (Figure 9H). It was also transported from the forepaws to the axillary lymph nodes in mice (Figure 9H). Notably, none of the *LEC* ^*ΔKras*^ mice exhibited chylous ascites or chylothorax. Together, these data suggest that loss of wild-type *Kras* in LECs does not affect the formation or function of lymphatic vessels.

## Discussion

KRAS^G12D^ expression during postnatal development was previously shown to cause the enlargement of lymphatic vessels and impair the development and maintenance of lymphatic valves (Homayun-Sepehr et al., 2021). In the present study, we show that hyperactive KRAS signaling in embryos impairs the morphogenesis of lymphovenous valves and causes lymphatic vessel hyperplasia. We also show that pharmacologic or genetic inhibition of MAPK activation can partially suppress lymphatic vessel hyperplasia in KRAS^G12D^-mutant embryos. These data extend our previous findings and indicate that KRAS/MAPK signaling must be tightly regulated during embryonic development for the proper development of lymphatic vessels.

The identification of causal genetic mutations for CLAs is facilitating the development of *in vitro* and *in vivo* models of CLAs. Recently, transient expression of KRAS^G12D^ or KRAS^G13D^ in LECs was reported to induce edema and the enlargement of lymphatic vessels in zebrafish larvae (Sheppard et al., 2023). Likewise, we show that KRAS^G12D^ expression in LECs causes edema and the enlargement of lymphatic vessels in mouse embryos. These findings highlight the similarities between the two model systems and suggest that KRAS serves a critical role in regulating the size of lymphatic vessels during embryonic development.

Lymphovenous valves are the first lymphatic valves to form, and they develop in a stepwise manner during embryonic development (Geng et al., 2016). Lymphovenous valve cells are specified at E12.0, begin to extend into veins at E12.5, and form well-defined valve leaflets by E14.5 (Geng et al., 2016). We found that E14.5 *iLEC* ^*KrasG12D*^ embryos lacked well-defined lymphovenous valve leaflets. The Ras pathway was previously shown to serve a critical role in the development of lymphovenous valves. The *Rasa1* gene encodes for p120, which is a Ras GTPase-activating protein (GAP) that negatively regulates RAS signaling by promoting GTP hydrolysis by RAS proteins (Chen et al., 2020). Conditional knockout of *Rasa1* in endothelial cells increases RAS/MAPK signaling and impairs the development of lymphovenous valves (Chen et al., 2020). Thus, our findings further highlight the importance of the RAS pathway in the formation of lymphovenous valves, which are essential for the normal physiology of the lymphatic system.

We found that the jugular lymph sacs of *LEC* ^*KrasG12D*^ embryos contained irregular clusters of LECs and hematopoietic cells. Similar cell clusters are present in *Prox1-11 kb* ^*Δ/Δ*^ embryos, which harbor a mutation in an enhancer upstream of *Prox1* (Kazenwadel et al., 2023). The cell clusters in *Prox1-11 kb* ^*Δ/Δ*^ embryos include Runx1-positive cells (Kazenwadel et al., 2023). Runx1 is expressed by hemogenic endothelium, a specialized endothelium that gives rise to hematopoietic cells (Kazenwadel et al., 2023). The discovery of Runx1-positive cells in *Prox1-11 kb* ^*Δ/Δ*^ embryos, and *ex vivo* colony-forming data, led investigators to propose that *Prox1* represses hemogenic activity by LECs (Kazenwadel et al., 2023). Like the clusters in *Prox1-11 kb* ^*Δ/Δ*^ embryos, the clusters in *LEC* ^*KrasG12D*^ embryos had Runx1-positive cells. This result raises the possibility that hyperactive KRAS signaling also induces hemogenic activity by LECs. However, a subset of hematopoietic stem cells come from Lyve1-positive cells (Lee et al., 2016). Thus, the irregular cell clusters in *LEC* ^*KrasG12D*^ embryos could be due to hyperactive KRAS signaling in the hematopoietic lineage. Future experiments with a more LEC-restricted Cre (e.g., *Prox1-CreER* ^*T2*^) are required to determine whether Kras^G12D^ signaling in LECs or the hematopoietic lineage triggers the formation of hematopoietic cell clusters in jugular lymph sacs.

Mutant LECs have been isolated from fluid collected from patients with macrocystic lymphatic malformations and effusion fluid harvested from patients with chylothorax (Osborn et al., 2015; Glaser et al., 2018; Li et al., 2023). These data suggest that malformations in patients shed LECs. Like patients, *LEC* ^*KrasG12D*^ embryos have free-floating LECs in their macrocystic malformations. The process by which mutant LECs delaminate from vessels is not understood. We found that KRAS^G12D^ expression in primary human LECs causes cells to adopt a mesenchymal morphology. This phenomenon has also been observed by others expressing RAS pathway mutations in endothelial cells (Nikolaev et al., 2018; Li et al., 2019; Boscolo et al., 2022; Sheppard et al., 2023). Our bulk RNA-Seq data shows that KRAS^G12D^-LECs express genes associated with epithelial-to-mesenchymal transition (EMT). Cells undergoing EMT lose their cell-cell contacts and gain migratory and invasive capabilities. EMT is driven by specific transcription factors, including *FOXC2*, *Twist*, *Zeb1*, *SNAI1* (Snail), and *SNAI2* (Slug). Of interest, we found that *SNAI2* was approximately 20-fold higher in KRAS^G12D^-LECs compared to GFP-LECs. *SNAI1* and *ZEB1* were also elevated in KRAS^G12D^-LECs. These results raise the possibility that EMT could drive the delamination of mutant LECs into the lumens of lymphatic vessels and cysts. Future studies aimed at blocking *SNAI2* or other EMT-inducing transcription factors could reveal the role of this process in the pathogenesis of complex lymphatic anomalies and other vascular malformations.

During embryonic and postnatal development, subsets of lymphatic vessels mature into collecting lymphatic vessels. During this maturation phase, lymphatic vessels recruit lymphatic muscle cells and acquire valves. The lymphovenous junction also matures and remodels to have valves. We previously showed that hyperactive Kras signaling in LECs affects the maturation of lymphatic vessels (Homayun-Sepehr et al., 2021). We found that Kras^G12D^ suppressed the recruitment of lymphatic muscle cells to lymphatic vessels and the formation of lymphatic valves (Homayun-Sepehr et al., 2021). Here, we show that Kras^G12D^ also affects the formation of lymphovenous valves. Our bulk transcriptome analysis of KRAS^G12D^-LECs and GFP-LECs shows that KRAS^G12D^ decreases the expression of genes involved in the recruitment of lymphatic muscle cells and the development of lymphatic valves. Additionally, we demonstrate that trametinib increases the expression of several of these genes in KRAS^G12D^-LECs. These results suggest that one mechanism by which hyperactive KRAS/MAPK signaling impairs the maturation of lymphatic vessels is by suppressing the expression of genes critical to this process. However, the downstream transcription factors mediating these changes in global gene expression remain unclear.

The discovery of activating RAS/MAPK pathway mutations in CLA patients has sparked interest in repurposing MEK1/2 inhibitors for CLAs. Of the FDA-approved MEK1/2 inhibitors, trametinib has been the most widely used in CLA patients and is reported to reduce patient symptoms (Li et al., 2019; Foster et al., 2020; Chowers et al., 2022). We previously reported that trametinib suppresses the loss of lymphatic valves in Kras^G12D^-mutant mice during postnatal development (Homayun-Sepehr et al., 2021). In the present study, we characterized the effect of trametinib on dermal lymphatic vessels in *LEC* ^*KrasG12D*^ embryos. We focused on dermal lymphatic vessels because the diameter of these vessels was dramatically increased in *LEC* ^*KrasG12D*^ embryos, with some areas appearing as continuous lymphatic sheets. Additionally, CLA patients with RAS pathway mutations have dilated lymphatic vessels (Liu et al., 2022). We found that blocking ERK1/2 activation with trametinib significantly decreased the diameter of lymphatic vessels in *LEC* ^*KrasG12D*^ embryos. Similarly, suppressing ERK1/2 activation by expressing a dominant-negative form of MEK1 (Map2k1^K97M^) in LECs also decreased the diameter of lymphatic vessels in *LEC* ^*KrasG12D*^ embryos. However, lymphatic vessels in trametinib-treated *LEC* ^*KrasG12D*^ embryos and *LEC* ^*KrasG12D;Map2k1K97M*^ embryos were still larger than normal lymphatic vessels. This could be due to incomplete inhibition of the MAPK pathway by trametinib or Map2k1^K97M^. Alternatively, sustained or enhanced PI3K/AKT signaling in the face of MEK1/2 inhibition could cause the enlargement of lymphatic vessels in *LEC* ^*KrasG12D*^ embryos. Future experiments conditionally knocking out *Map2k1*/*Map2k2* (MEK1 and MEK2) in *LEC* ^*KrasG12D*^ mice or combining PI3K inhibitors (e.g., alpelisib) with trametinib could help distinguish between these possibilities.

KRAS, HRAS, and NRAS are highly related small GTPases encoded by three separate genes and somatic activating mutations in these genes have been identified in complex lymphatic anomaly patients (Manevitz-Mendelson et al., 2018; Barclay et al., 2019; Ozeki et al., 2019; Nozawa et al., 2020; Homayun-Sepehr et al., 2021; Li et al., 2023; Sheppard et al., 2023). This has fueled intense research efforts to identify the mechanisms by which mutant RAS genes cause lymphatic anomalies. Despite the growing interest in the function of mutant RAS genes in LECs, relatively little is known about the role of wild-type *KRAS*, *HRAS*, and *NRAS* in the development of the lymphatic system. *Kras* knockout mice die during embryonic development, whereas *Hras* and *Nras* knockout mice are viable (Ichise et al., 2010). Therefore, to investigate the function of RAS genes in the development of the lymphatic system, various *Kras*, *Hras*, and *Nras* compound mutant lines of mice were generated and analyzed (Ichise et al., 2010). This revealed that mice heterozygous for *Kras* and lacking either *Hras* or *Nras* develop chylous ascites and lymphatic hypoplasia (Ichise et al., 2010). However, the effect of the compound mutations on collecting vessels, valves, and lymphatic function was not delineated, and the specific role *Kras* serves in the development of lymphatic vessels was not investigated. We found that conditional deletion of *Kras* in LECs did not affect the formation of lymphatic capillaries, collecting lymphatic vessels, or valves. Interestingly, our transcriptome analysis of primary human LECs shows that LECs express *HRAS* and *NRAS*. Therefore, the lack of a lymphatic phenotype in *LEC* ^*ΔKras*^ mice could be due to compensation by *Hras* or *Nras*. Together, our results suggest that wild-type *Kras* is dispensable for the development and function of lymphatic vessels.

In conclusion, we show that hyperactive KRAS signaling in LECs during embryonic development causes lymphatic vessel defects. We also demonstrate that inhibition of the MAPK pathway can suppress changes to cellular processes and lymphatic vessel growth induced by mutant KRAS. Together, these data further support the testing of MEK1/2 inhibitors for the treatment of complex lymphatic anomalies.

## Methods

### Mice and genotyping

The animal experiments described in this manuscript were carried out in accordance with an animal protocol approved by the Institutional Animal Care and Use Committee of UT Southwestern Medical Center. Mice were maintained in ventilated microisolator cages and were fed a standard diet. Mice were provided igloos and nestlets as enrichment items. Male and female mice were used in experiments. *Lyve1-Cre*, *LSL-Kras* ^*G12D*^, *LSL-Map2k1* ^*K97M*^, and *Kras* ^*loxp*^ mice were previously described (Jackson et al., 2001; Kelleher et al., 2004; Pham et al., 2010; Damnernsawad et al., 2016).

### Trametinib preparation

For *in vitro* experiments, trametinib (GSK1120212; 5 mg; Selleck, S2673) was dissolved in DMSO (Sigma-Aldrich, D8779) to create a 10 µM stock solution. We used a final concentration of 10 nM for all *in vitro* experiments. For *in vivo* experiments, we first dissolved trametinib (5 mg) in DMSO (1 mL; Sigma-Aldrich, D8779). We then added PEG300 (4 mL; Sigma-Aldrich, 8.07484), Tween 80 (500 μL; Sigma-Aldrich, P4780), and saline (4.5 mL; Baxter, 2F7124). Vehicle or trametinib (1 mg/kg) was administered to mice with a 20-gauge gavage needle.

### Primary antibodies

The following primary antibodies were used for immunoblotting or immunofluorescence staining: goat anti-Lyve1 (R&D Systems, #AF2125; dilution 1:250), rabbit anti-phospho AKT (Ser 473, Cell Signaling, 4060S; dilution 1:1000), rabbit anti-AKT (Cell Signaling, 4685; dilution 1:1000), rabbit anti-phospho ERK1/2 (Cell Signaling, 4370; dilution 1:1000), rabbit anti-ERK1/2 (Cell Signaling, 4695; dilution 1:1000), rabbit anti-tubulin (Cell Signaling, 2148; dilution 1:2000), rabbit anti-GAPDH (Cell Signaling, 2118; dilution 1:1000), chicken anti-GFP (abcam ab13970; 1:4000), rat anti-endomucin (Santa Cruz, sc-65495; dilution 1:50), rabbit anti-Prox1 (AngioBio, 11-002; dilution 1:1000), rabbit anti-neuropilin-2 (Cell Signaling, 3366; dilution 1:500), rabbit anti-Runx1 (Abcam, ab92336; dilution 1:1000), and rabbit anti-KRAS^G12D^ (Cell Signaling, #14429; dilution 1:1000).

### Immunofluorescence staining of tissue sections

Slides were deparaffinized with xylene and rehydrated through a descending ethanol series. Non-specific binding was blocked by incubating slides with TBST +3% donkey serum for 30 min. Slides were then incubated overnight with primary antibodies diluted in TBST +5% BSA. Slides were washed with TBST and then incubated with fluorophore-conjugated secondary antibodies diluted in TBST +5% BSA. Following washes with TBST, coverslips were mounted with ProLong Gold + DAPI (Invitrogen, #36935).

### Whole-mount immunofluorescence staining

Back skin was harvested from E14.5 and E15.5 embryos for whole-mount immunofluorescence staining. Fixed samples were washed with PBS, permeabilized with PBS +0.3% TX-100 (PBST), and then blocked overnight with PBST +3% donkey serum. Samples were then incubated overnight with primary antibody diluted in PBST. After washing samples with PBST (3 × 40 min), they were incubated overnight with a secondary antibody diluted in PBST. Following washes with PBST (3 × 40 min), samples were placed on glass slides, and coverslips were mounted with ProLong Gold (Invitrogen, #36934).

### Analysis of lymphatic branch points and vessel diameters

*Analysis of back skin whole-mounts:* Images of back skin were captured at ×10 magnification, and the number of lymphatic branch points was manually counted. The same images were used to measure vessel diameters. We placed a 12 × 12 grid over the images and measured the diameter of vessels located on intersecting grid lines. We used NIS Elements software (version 5.30.02) to assess branch points and diameters.

#### Analysis of ear skin whole-mounts

To assess branch points, we manually counted the number of lymphatic branch points per 10X image. To measure vessel diameters, we placed a 12 × 12 grid over images and measured the diameter of vessels located on intersecting grid lines. To assess lymphatic valves per mm vessel, we measured the length of the lymphatic network and then manually counted the number of lymphatic valves. We used NIS Elements software (version 5.30.02) to manually count branch points and valves and to measure vessel lengths and diameters.

### Tissue culture and lentivirus treatment

Primary human LECs were purchased from PromoCell (C-12216) and cultured in EGM-2MV media (LONZA CC-3125). Cells were used between passages 3 and 6. GFP-expressing, KRAS^WT^-expressing, and KRAS^G12D^-expressing lentivirus particles were purchased from Vector Builder. We expressed the 4A-splice variant of *KRAS* (*KRAS4A*). To infect cells, we treated LECs with polybrene (final concentration 8 μg/mL) and lentivirus particles (MOI = 12.5). We selected an MOI of 12.5 because we found that the infection efficiency of the CMV-GFP lentivirus was greater than 90% when we used an MOI of 12.5 (data not shown). The tissue culture media was replaced with normal growth media 24 h after infecting the cells.

#### Proliferation/viability assays

To assess cell proliferation/viability, we plated LECs (3,000 cells per well) into the wells of a 96-well plate. Cells were infected with lentivirus the following day. Twenty-4 hours later, we replaced the media with regular growth media or with growth media containing DMSO or trametinib (10 nM). For experiments with regular growth media, we measured proliferation/viability 72 h after treating cells with lentivirus. For experiments with DMSO and trametinib, we measured proliferation/viability 72 h after treating cells with DMSO or trametinib. To measure proliferation/viability, we added MTS reagent (abcam, #ab197010) to the wells and read absorbance (490 nm) with a plate reader.

#### Scratch assays

LECs were plated into the wells of a 6-well or 12-well plate (50,000 cells/ml). Cells were infected with lentivirus the following day. Twenty-4 hours later, we replaced the media with regular growth media and cultured the cells until confluent. We used a p200 pipet tip to wound the monolayers. We then replaced the media with regular growth media or with growth media containing DMSO or trametinib (10 nM) and immediately imaged the cells. We reimaged the cells approximately 14 hours later. FIJI (Figi Is Just ImageJ) was used to measure the wounded area at both timepoints. Mitomycin C was not added to the scratch assays. Therefore, cell proliferation could contribute to the closure of the wounded areas.

### Immunoblotting

Cells were scraped in lysis buffer [mPER (Thermo Scientific, #78501) +Protease Inhibitor (Thermo Scientific, #78425) +Phosphatase Inhibitors I and II (Sigma-Aldrich, P2850 and P5726)], spun for 10 min at 4°C, and then supernatants were transferred to new tubes. Protein concentrations were determined with a BCA kit (Thermo Scientific, #23227). Proteins were separated by SDS-PAGE and then transferred to PVDF membranes. Non-specific binding was blocked by incubating membranes with TBST +5% non-fat milk for 30 min. Membranes were then incubated overnight with primary antibodies. After washing with TBST, membranes were incubated with the appropriate HRP-conjugated secondary antibodies. Bound antibodies were detected with SuperSignal West Dura Extended Duration Substrate (Thermo Scientific, #34075).

### ELISAs

We used commercially available ELISA kits to measure VEGF-C (R&D Systems, DY752B) and phospho-VEGFR3 (R&D Systems, DYC2724-2) levels. We measured VEGF-C levels in cell lysates and conditioned media from GFP-LECs and KRAS^G12D^-LECs. Phospho-VEGFR3 levels were measured using lysates from unstimulated cells and cells stimulated for 10 min with recombinant VEGF-C (R&D Systems, 9199-VC-025-CF; final concentration = 400 ng/mL).

### RNA isolation, sequencing, and bioinformatic analysis

To examine the effect of hyperactive KRAS signaling on gene expression, primary human LECs were infected with lentivirus particles, as described above. Twenty-4 hours later, the media was replaced with standard growth media. RNA was isolated with a Qiagen RNeasy Plus Mini Kit (Qiagen, #74314) 72 h after infecting the cells. To assess the impact of trametinib on KRAS^G12D^-LECs, primary human LECs were infected with lentivirus particles, and the media was replaced with normal growth media 24 h later. Forty-8 hours after infecting cells, they were treated with DMSO or trametinib (10 nM) for approximately 16 h. RNA was then isolated with a Qiagen RNeasy Plus Mini Kit (Qiagen, #74314). Samples were submitted to the Next-Generation Sequencing Core at UT Southwestern Medical Center for library preparation with the TruSeq RNA library prep kit and sequencing with an Illumina NextSeq 2000 system. Gene expression analyses were performed using Partek Flow software. Single-end reads were aligned to the human reference genome (hg38) using STAR, and gene expression was quantified using Ensembl (v109) protein-coding transcript annotations. Transcripts with no detectable expression in any sample were excluded. Expression counts were normalized using the Counts per Million mapped reads (CPM) method for subsequent differential gene expression analysis using the Partek Gene Specific Analysis (GSA) method. GO term analysis was performed with DAVID (https://david.ncifcrf.gov). Transcription regulatory networks were identified with Metascape (www.metascape.org). GraphPad Prism software (Version 9.5.1) was used to generate volcano plots and heat maps.

### CD31-positive cell isolation and reverse-transcription PCR

Mice were euthanized, and lungs were perfused with PBS + heparin. Lungs were dissected, minced with a scalpel, placed in a gentleMACS C tube (Miltenyi Biotec, #130-093-237), and then digested with a mouse lung dissociation kit (Miltenyi Biotec, #130-095-927). A gentleMACS tissue dissociator was used to create a single-cell suspension. CD31 microbeads (Miltenyi Biotec, #130-097-418), MS columns (Miltenyi Biotec, 130-042-201), and a miniMACS separator (Miltenyi Biotec, #130-090-312) were used according to the manufacturer’s instructions to isolate CD31-positive endothelial cells. RNA was isolated with TRIzol, and cDNA was generated with an iScript cDNA synthesis kit (Bio-Rad, #1708890). The following primers were used to detect *Kras* 5′-TGA​GTA​TAA​ACT​TGT​GGT​GGT​TGG​A-3′ and 5′-TCT​ATC​GTA​GGG​TCA​TAC​TCA​TCC-3’. The following primers were used to detect *Gapdh* 5′-AGG​TCG​GTG​TGA​ACG​GAT​TT-3′ and 5′-ACT​GTG​CCG​TTG​AAT​TTG​CC-3’.

### Evans blue dye lymphangiography

Mice were anesthetized with an intraperitoneal injection of avertin. Evans blue dye (EBD) (1% w/v) was serially injected intradermally into the hind paws and forepaws of control and mutant mice. Approximately 5 min later, the iliac, thoracic, and axillary regions were dissected and visualized under a dissecting microscope.

### Microscope and camera information

Back skin samples and fluorescent tissue sections were imaged with a Nikon DS-Qi2 camera attached to a Nikon Eclipse E600 microscope. Tissue sections stained by immunohistochemistry were imaged with an AmScope MU1000 camera attached to a Nikon Eclipse E600 microscope. Embryos were imaged with an AmScope MU1000 camera attached to an AmScope dissecting microscope. Cells were imaged with an AmScope MU1000 camera attached to a Nikon Eclipse TS100 microscope.

### Statistical analysis

Data were analyzed using GraphPad Prism statistical analysis software (Version 9.5.1). All results are expressed as mean ± SEM. The number of mice in each group is indicated in the figure legends (n = number of mice). For experiments with two groups, unpaired Student’s *t*-tests were performed to test means for significance. For experiments with more than two groups, group differences were assessed by ANOVA followed by Dunnett’s multiple comparisons test or Tukey’s multiple comparisons test. We performed Fisher’s exact tests to analyze contingency tables for the incidence of edema, lymphovenous valves, and cell clusters in *LEC* ^*Ctrl*^ and *LEC* ^*KrasG12D*^ embryos. Data were considered significant at *p* < 0.05.

## Data Availability

The datasets presented in this study can be found in online repositories. The names of the repository/repositories and accession number(s) can be found below: https://www.ncbi.nlm.nih.gov/geo/, GSE239737.

## Supplementary material

Supplemental Figure S1 Western blot results for phospho-AKT, AKT, phospho-ERK1/2, ERK1/2, and GAPDH. Protein lysates were made 72 hours after treating primary human LECs with GFP, KRAS^WT,^ or KRAS^G12D^-expressing lentivirus particles.

Supplemental Figure S2 VEGF-C/VEGFR3 signaling in KRAS^G12D^-LECs. **(A)**. VEGF-C ELISA results using lysates from GFP-LECs and KRAS^G12D^-LECs. **** *P* < 0.0001; unpaired Student’s *t*-tests. **(B)**. VEGF-C ELISA results using conditioned media from GFP-LECs and KRAS^G12D^-LECs. **** *P* < 0.0001; unpaired Student’s *t*-tests. **(C)**. Phospho-VEGFR3 ELISA results for unstimulated and VEGF-C stimulated (10 minutes, 400 ng/ml) GFP-LECs and KRAS^G12D^-LECs. **** *P* < 0.0001; ANOVA Tukey’s multiple comparisons test. **(D)**. Western blot analysis of phospho-AKT, AKT, phospho-ERK1/2, ERK1/2, and tubulin. Cells were grown overnight in reduced-serum media before being stimulated with recombinant VEGF-C (10 minutes, 400 ng/ml). **(E)**. Graphs of western blot results. ** *P* < 0.01; **** *P* < 0.0001; ANOVA Tukey’s multiple comparisons test.

Supplemental Figure S3 Transcription regulatory networks altered by KRAS^G12D^ and trametinib. **(A,B)**. Genes upregulated **(A)** or down-regulated **(B)** by KRAS^G12D^ were subjected to motif enrichment analysis. Several of the top transcription factors from the analysis are shown. **(C,D)**. Genes upregulated **(C)** or down-regulated **(D)** by trametinib in KRAS^G12D^-LECs were subjected to motif enrichment analysis. Several of the top transcription factors from the analysis are shown.

Supplemental Figure S4
**(A–D)**. Low magnification images of back skin from E14.5 embryos stained for neuropilin-2. **(A’–D’)**. Zoomed in images of the boxed regions shown in panels A-D. Scale bar = 500 µm.

Supplemental Figure S5
**(A–D)**. Low magnification images of back skin from E15.5 embryos stained for neuropilin-2. **(A’–D’)**. Zoomed in images of the boxed regions shown in panels A-D. Scale bar = 500 µm.
