# Supplementary figures and images for: Hyperactive KRAS/MAPK signaling disrupts normal lymphatic vessel architecture and function
Source: Front Cell Dev Biol. 2023 Sep 25;11:1276333. doi: 10.3389/fcell.2023.1276333 (PMC10571159; doi:10.3389/fcell.2023.1276333)

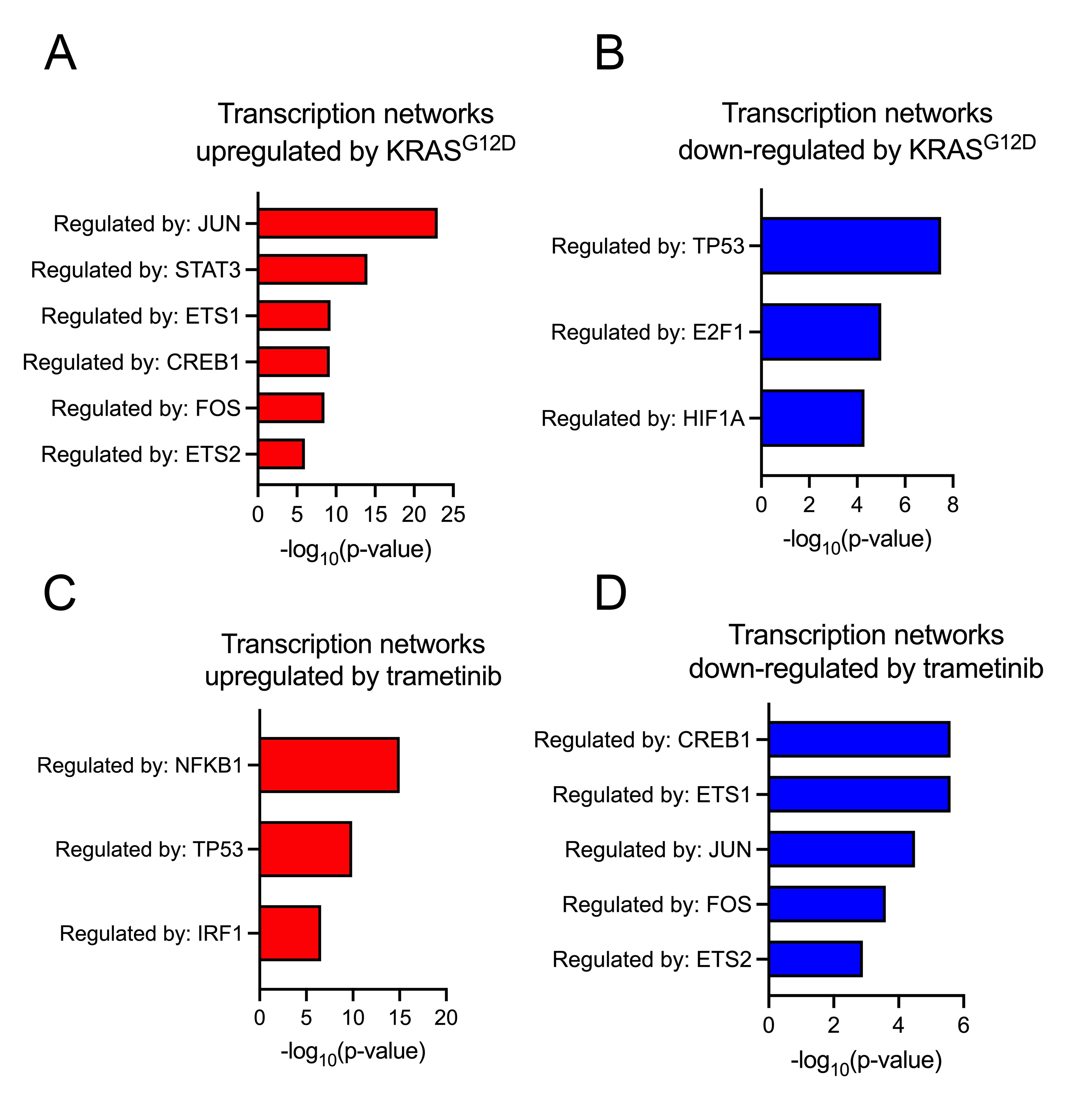

Supplement: Supplementary file 1 [file Image3.JPEG]

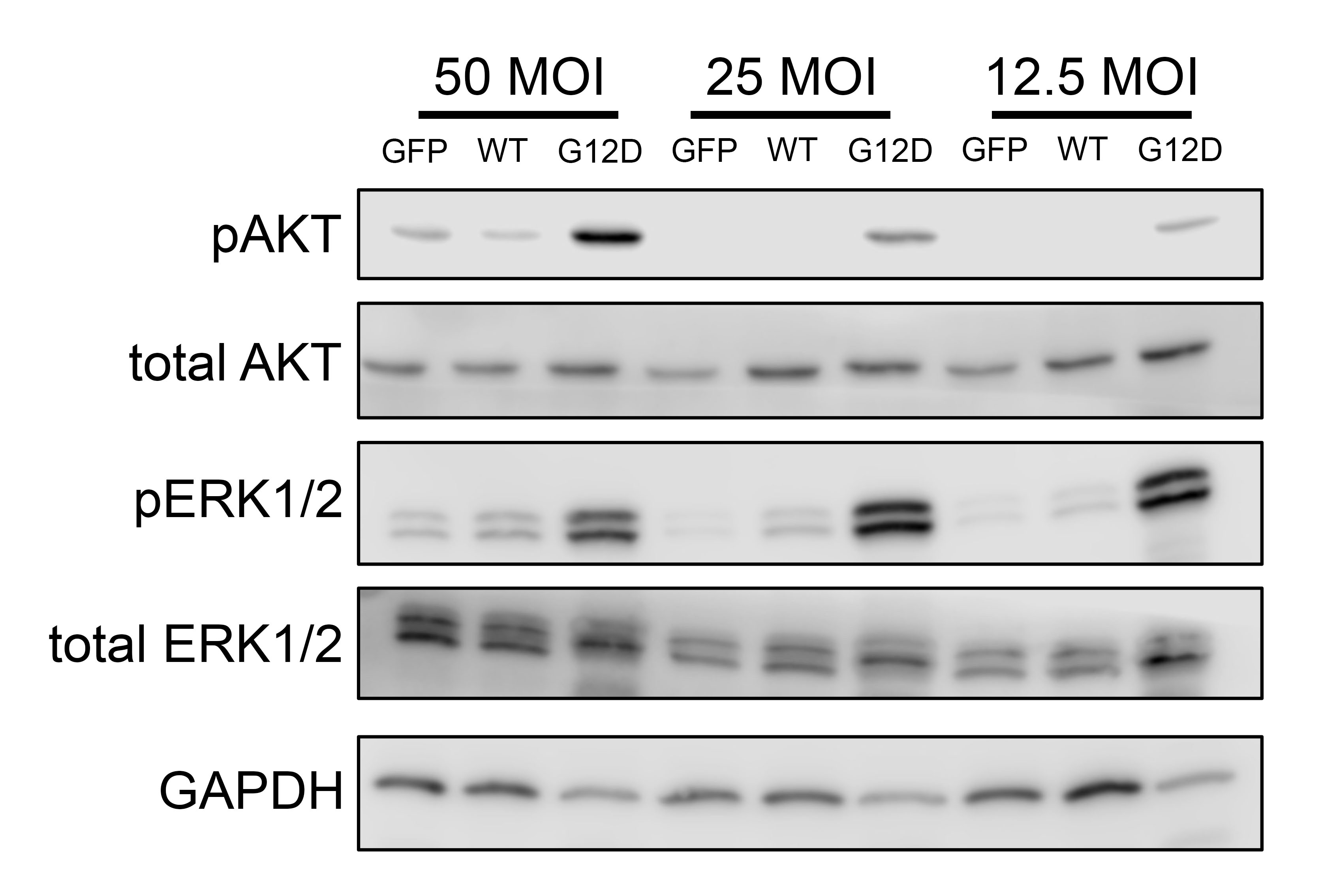

Supplement: Supplementary file 2 [file Image1.JPEG]

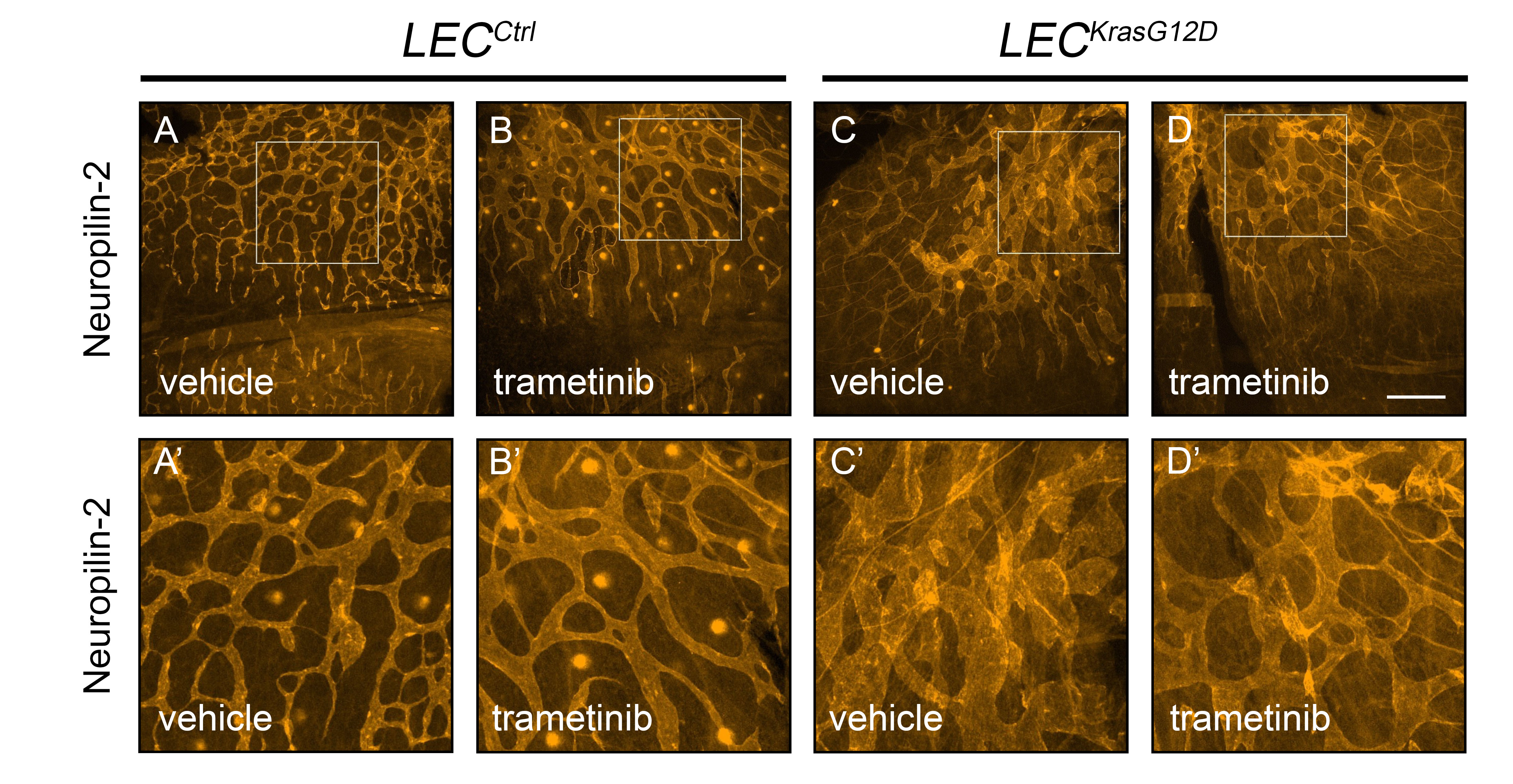

Supplement: Supplementary file 3 [file Image4.JPEG]

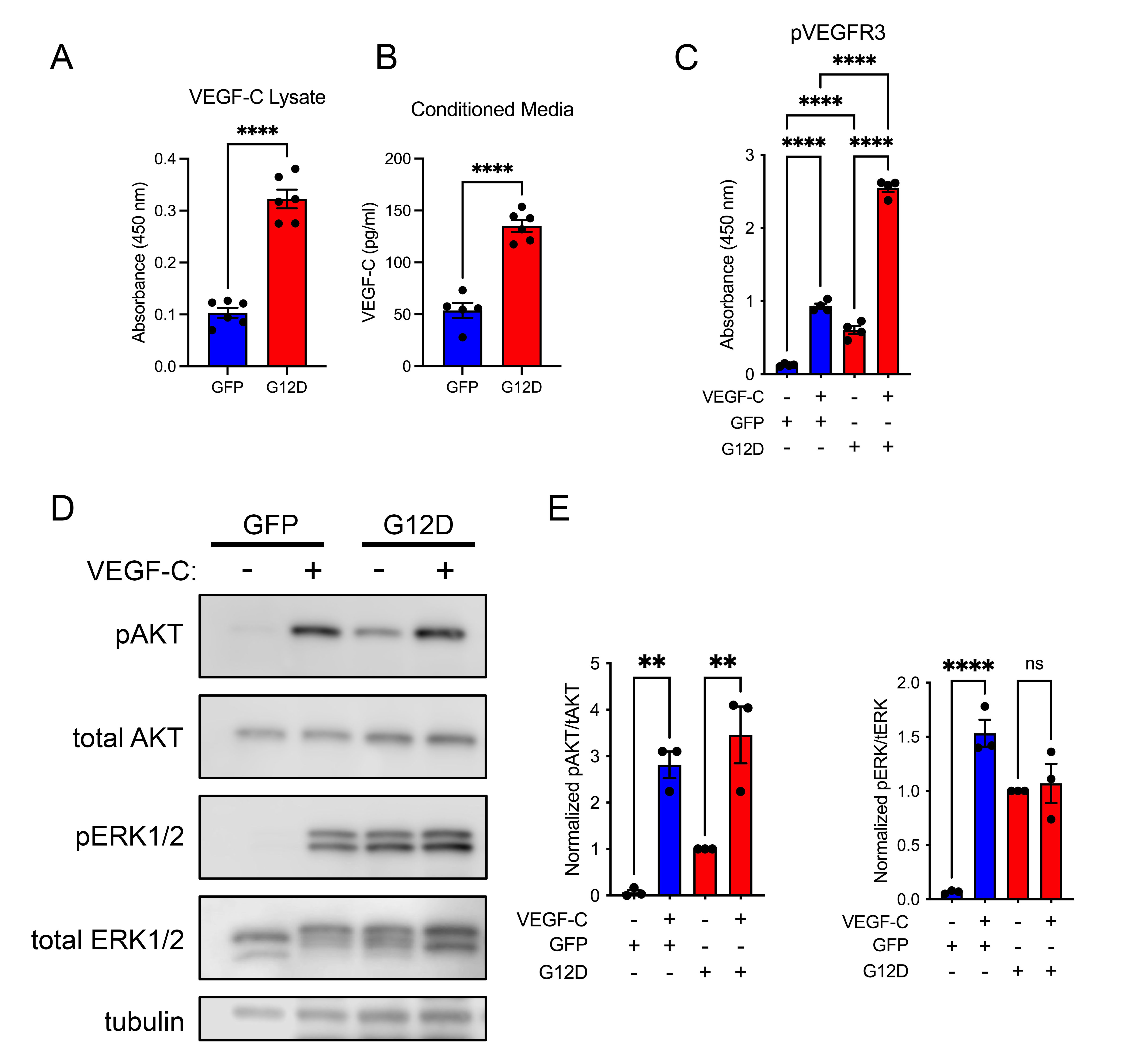

Supplement: Supplementary file 4 [file Image2.JPEG]

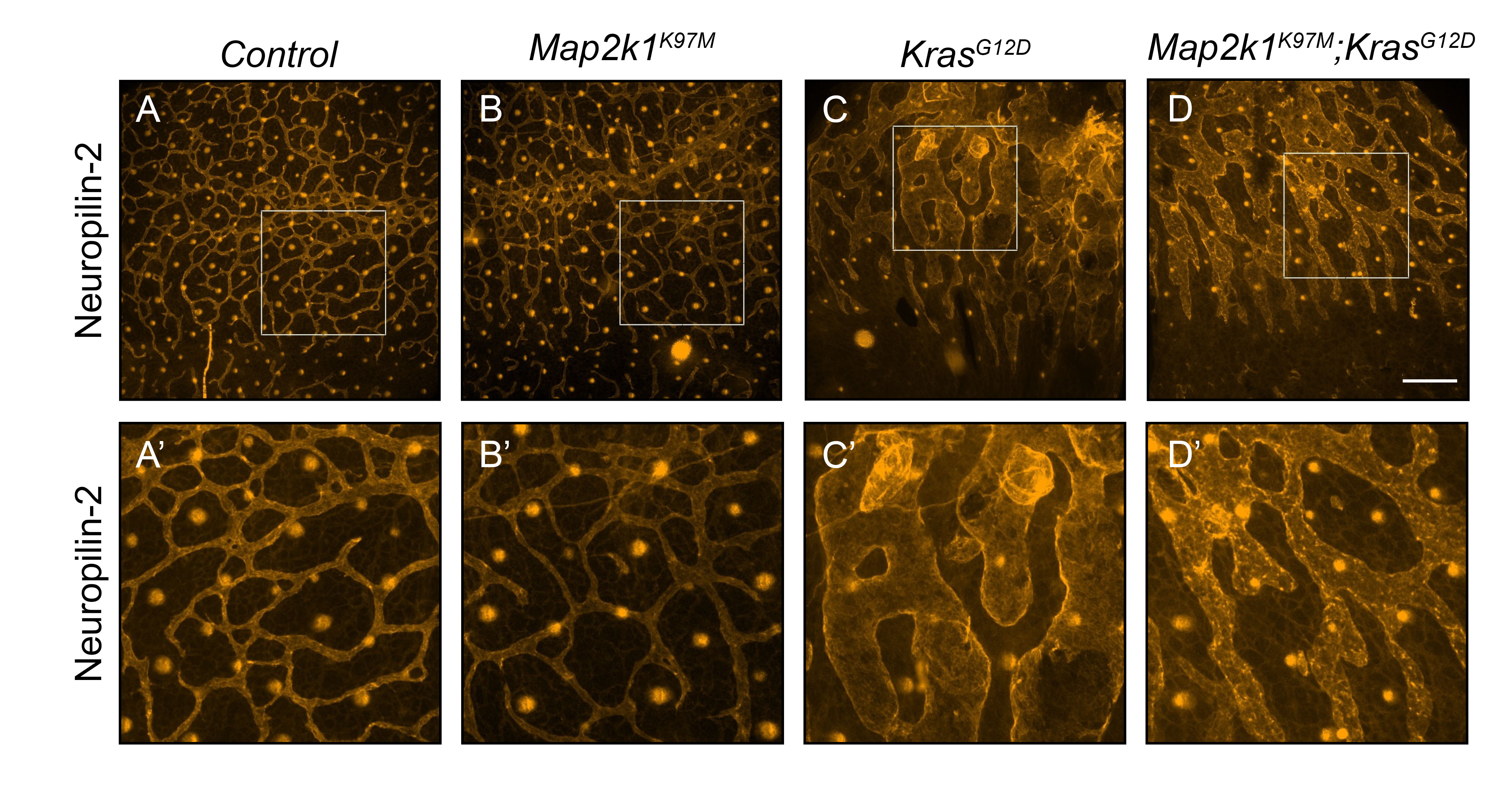

Supplement: Supplementary file 5 [file Image5.JPEG]
